# Observations on the Lovász *θ*-Function, Graph Capacity, Eigenvalues, and Strong Products †

**DOI:** 10.3390/e25010104

**Published:** 2023-01-04

**Authors:** Igal Sason

**Affiliations:** 1 Andrew & Erna Viterbi Faculty of Electrical and Computer Engineering, Technion—Israel Institute of Technology, Haifa 3200003, Israel; eeigal@technion.ac.il; Tel.: +972-4-8294699; 2 Faculty of Mathematics, Technion—Israel Institute of Technology, Haifa 3200003, Israel

**Keywords:** Lovász *θ*-function, Shannon capacity of a graph, strongly regular graph, strong product of graphs, vertex- and edge-transitivity, Alon–Boppana bound, Ramanujan graph, chromatic number

## Abstract

This paper provides new observations on the Lovász θ-function of graphs. These include a simple closed-form expression of that function for all strongly regular graphs, together with upper and lower bounds on that function for all regular graphs. These bounds are expressed in terms of the second-largest and smallest eigenvalues of the adjacency matrix of the regular graph, together with sufficient conditions for equalities (the upper bound is due to Lovász, followed by a new sufficient condition for its tightness). These results are shown to be useful in many ways, leading to the determination of the exact value of the Shannon capacity of various graphs, eigenvalue inequalities, and bounds on the clique and chromatic numbers of graphs. Since the Lovász θ-function factorizes for the strong product of graphs, the results are also particularly useful for parameters of strong products or strong powers of graphs. Bounds on the smallest and second-largest eigenvalues of strong products of regular graphs are consequently derived, expressed as functions of the Lovász θ-function (or the smallest eigenvalue) of each factor. The resulting lower bound on the second-largest eigenvalue of a *k*-fold strong power of a regular graph is compared to the Alon–Boppana bound; under a certain condition, the new bound is superior in its exponential growth rate (in *k*). Lower bounds on the chromatic number of strong products of graphs are expressed in terms of the order and the Lovász θ-function of each factor. The utility of these bounds is exemplified, leading in some cases to an exact determination of the chromatic numbers of strong products or strong powers of graphs. The present research paper is aimed to have tutorial value as well.

## 1. Introduction

The notion of the graph capacity in Shannon’s problem of zero-error communication [1] had a significant impact on the development of information theory and graph theory, including the introduction of perfect graphs by Berge [2], strong graph products (and strong graph powers) [3,4], the introduction of the Lovász θ-function of a graph as a computable upper bound on its Shannon capacity [5,6], the rank-bound by Haemers [7], and other important follow-up works that are surveyed, e.g., in [3,8,9,10].

In graph theory, there are four central sorts of graph products, each with its own applications and theoretical interpretations. The reader is referred to the excellent handbook [11], which presents the rich and fertile field of graph products. Strong product of graphs is one of the most extensively studied sorts of graph products, and there exists a polynomial-time algorithm that finds the unique prime factorization of any connected graph with that type of multiplication [12]. Strong powers of graphs are also fundamental in information theory. Their information-theoretic significance stems from the notion of the Shannon capacity of graphs for error-free communication [1], and the Witsenhausen rate [13] in the zero-error source coding problem with perfect side information at the receiver. Properties of strong products and strong powers of graphs, and bounds on their independence numbers and chromatic numbers have been extensively studied, e.g., in [1,3,4,6,7,8,9,11,12,13,14,15,16,17,18,19,20,21,22,23,24,25,26,27,28,29,30,31,32,33,34,35,36,37,38].

The present work continues the above paths of research. It provides some new observations on the Lovász θ-function of regular graphs, calculation of the Shannon capacity of some strongly regular graphs, bounds on eigenvalues of graphs (in particular, the second-largest and smallest eigenvalues of the adjacency matrix, which play a key role in spectral graph theory), bounds related to Ramanujan graphs, and strong products of graphs. The analysis in the present work mainly relies on the notion of the Lovász θ-function of graphs [6]. The paper includes a thorough review of the backgrounds relevant to this work with suitable references or explanations, which also serve to motivate the presentation of the results in this work and to put them into perspective. The presentation in this research paper is consequently aimed to have tutorial value as well.

The results obtained in this work are outlined as follows:

(1) A known upper bound on the Lovász θ-function of a regular graph is expressed in terms of the smallest eigenvalue of its adjacency matrix [6]. A key result in this work provides a lower bound on the Lovász θ-function of a regular graph, which is expressed in terms of the second-largest eigenvalue of its adjacency matrix. New sufficient conditions for equalities in these bounds are also obtained (Proposition 1).
(2) A simple and closed-form expression of the Lovász θ-function is derived for all strongly regular graphs (Corollary 1).
(3) Eigenvalue inequalities are derived, which relate the smallest and second-largest eigenvalues of a regular graph. They hold with equality if and only if the graph is strongly regular (Corollaries 2 and 3).
(4) The Shannon capacity of several strongly regular graphs is determined (Section 3.5).
(5) Bounds on parameters of regular graphs, and in particular of Ramanujan graphs, are derived (Corollaries 4–6).
(6) Bounds on the smallest and the second-largest eigenvalues of strong products of regular graphs are derived, which are expressed in terms of calculable parameters of its factors (Proposition 2).
(7) A new lower bound on the second-largest eigenvalue of a *k*-fold strong power of a regular graph is compared to the Alon–Boppana bound. Under a certain condition, the former bound shows an improvement in its exponential growth rate as a function of *k* (Section 3.3).
(8) Every non-complete and non-empty connected regular graph, whose Lovász θ-function is below a certain value, is proved to have the property that almost all its strong powers are highly non-Ramanujan (Proposition 3).
(9) Lower bounds on the chromatic number of strong products of graphs are expressed in terms of the order and Lovász θ-function of each factor (Proposition 4). Their utility is exemplified, while also leading to exact chromatic numbers in some cases.

The paper is structured as follows: Section 2 provides notation and preliminary material that is essential for this paper. Section 3 provides the results of this work, followed by examples and discussions. It is composed of five subsections that address issues related to the Lovász θ-function, Shannon capacity of graphs, Ramanujan graphs, the second-largest and smallest eigenvalues of strong products or strong powers of graphs, and the chromatic numbers of such graphs. Section 4 proves the results in Section 3.

## 2. Preliminaries

This section provides essential notation and preliminaries for this paper. The following standard notation in set theory is used: N={1,2,…} is the set of natural numbers, R is the set of real numbers, and [n]≜{1,…,n} with n∈N. The cardinality of a set A is a measure of its number of elements; it is denoted by |A|, and (by definition) it is equal to its number of elements if A is a finite set.

Let G be a graph, and let V(G) and E(G) denote, respectively, the sets of vertices and edges in G. The *order* and *size* of a graph G are defined to be |V(G)| and |E(G)|, respectively. A graph G is said to be *finite* if V(G) is a finite set. A pair of vertices are *adjacent* in a graph G if these two vertices are the endpoints of an edge e∈E(G).

A graph is called *simple* if it has no loops (i.e., it has no edge with identical endpoints), and if there are no multiple edges between any pair of adjacent vertices. A graph G is said to be *undirected* if its edges have no directions; otherwise, it is a directed graph, also known as a *digraph*. Throughout this paper, it is assumed that the graphs under consideration are finite, undirected, and simple.

An *empty graph* is an edgeless graph. A graph G is said to be an *r-partite graph* if its vertex set V(G) is a disjoint union of *r* subsets such that every pair of vertices that are elements of an identical subset are non-adjacent. If r=2, then it is a *bipartite graph*.

A *walk* in a graph G is a sequence of its vertices such that (by definition) every pair of consecutive vertices are adjacent. A *path* in a graph is a walk with no repeated vertices (in other words, a path is a walk along the vertices of G such that no vertex can be visited twice). The *length of a path* is defined as its number of edges. Hence, $P=[v_{1},\ldots ,v_{\ell }]$ is a path in a graph G if $\left\{v_{i},v_{i+1}\right\}\in E\left(G\right)$ for all i∈[ℓ−1], and all the vertices in the sequence ${\left\{v_{i}\right\}}_{i=1}^{\ell }$ are distinct; the endpoints of the path P are $v_{1}$ and $v_{\ell }$, and its length is equal to ℓ−1. A *cycle* C in a graph G is obtained by adding an edge to a path P such that it gives a closed walk (i.e., a walk whose endpoints are identical). The cycle $C=[v_{1},\ldots ,v_{\ell },v_{1}]$ is of length *ℓ*, which is obtained by adding the edge $e=\left\{v_{\ell },v_{1}\right\}\in E\left(G\right)$ to the above (ℓ−1)-length path P; the two identical endpoints of the cycle C are the vertex $v_{1}$.

A graph is said to be *connected* if every two distinct vertices in that graph are connected by a path (otherwise, it is a *disconnected graph*). The *distance* between a pair of distinct and connected vertices in a graph is defined to be the length of the shortest path whose two endpoints are the given pair of vertices. The distance between two disconnected vertices in a graph is defined to be infinite, and the distance between a vertex and itself is set to be zero. The *diameter* of a graph is the maximum distance between any pair of vertices in that graph. A connected and finite graph has a finite diameter, and the diameter of a disconnected graph is (by definition) infinite.

The following standard notation in graph theory is used:

(a) $K_{n}$ denotes the *complete graph* with n∈N vertices, where every pair of distinct vertices are adjacent; hence, $K_{1}$ is an empty graph with a single vertex.
(b) $K_{n,m}$ denotes the *complete bipartite graph*, which is a bipartite graph consisting of a vertex set that is a disjoint union of two finite sets $V_{1}$ and $V_{2}$ of cardinalities *n* and *m*, respectively, and a set of edges that are all the possible connections of a vertex in $V_{1}$ and a vertex in $V_{2}$.
(c) $P_{n}$ denotes an *(n−1)-length path* with n∈N, which is a graph with *n* vertices that forms a path of length n−1; in particular, $P_{1}=K_{1}$.
(d) $C_{n}$ denotes an *n-length cycle*, which is a graph with n≥3 vertices that forms a cycle of length *n*.
(e) K(m,r) denotes the *Kneser graph* with integers 1≤r≤m. It has n=mr vertices, represented by all *r*-subsets of [m]. Two vertices are adjacent in that graph if they are represented by disjoint *r*-subsets. The graph K(m,r), provided that it has more than one vertex, is a connected graph if and only if either m>2r or (m,r)=(2,1).

A *subgraph* is a graph that exists within another graph. More formally, F is a subgraph of a graph G if V(F)⊆V(G) and E(F)⊆E(G). If F is a subgraph of G, we write F⊆G. A *spanning subgraph* is obtained by edge deletions from the original graph, while its vertex set is left unchanged. An *induced subgraph* is obtained by removing vertices from the original graph, followed by the deletion of the edges that are adjacent to these removed vertices.

A *clique* in a graph G is a subset of pairwise adjacent vertices in G (in other words, the induced subgraph of G on that subset is a complete subgraph). The maximum size of a clique in G is called the *clique number* of G, and it is denoted by ω(G). Similarly, an *independent set* (also known as a *coclique*) in G is a subset of pairwise non-adjacent vertices. The maximum size of an independent set is called the *independence number* of G, and it is denoted by α(G). A *proper vertex coloring* of G is a coloring of its vertices such that no pair of adjacent vertices are assigned the same color. The smallest required number of colors for a proper coloring of the vertices in G is called the *chromatic number* of G, and it is denoted by χ(G).

Let G=(V(G),E(G)) be a finite, undirected, and simple graph of order |V(G)|=n. Define the *adjacency matrix* A=A(G) of the graph G to be an *n*-times-*n* symmetric matrix such that $A={\left(a_{i,j}\right)}_{1\leq i,j\leq n}$ with $a_{i,j}=1$ if {i,j}∈E(G), and $a_{i,j}=0$ otherwise (hence, the diagonal elements of A are in particular zeros). Let the eigenvalues of A (also known as the eigenvalues of G) be given in decreasing order by

(1) $$\begin{array}{c} \lambda_{\max}\left(G\right)=\lambda_{1}\left(G\right)\geq \lambda_{2}\left(G\right)\geq \ldots \geq \lambda_{n}\left(G\right)=\lambda_{\min}\left(G\right). \end{array}$$

The *spectrum* of G consists of the eigenvalues of A, including their multiplicities. The terms $\lambda_{1}\left(G\right),\lambda_{2}\left(G\right)$, and $\lambda_{n}\left(G\right)$ are referred to as the *largest, second-largest, and smallest eigenvalues* of the graph G, respectively. The second-largest and smallest eigenvalues play a key role in *spectral graph theory*, and the interested reader is referred to, e.g., [39,40,41,42,43].

The number of edges that are incident to a vertex in a graph is called the *degree* of the vertex, and a graph is said to be *regular* if its vertices have an identical degree. A regular graph whose all vertices have a fixed degree *d* is called a *d-regular* graph, and it is said to have *valency d*. A *d*-regular graph has a largest eigenvalue $\lambda_{1}\left(G\right)=d$, with the all-ones column vector (of length *n*) as an eigenvector.

Let G be a finite, simple and undirected graph. The *complement* of G, denoted by $\bar{G}$, is defined to have the same vertex set as G, and (by definition) any pair of distinct vertices in V(G) are adjacent in $\bar{G}$ if and only if they are non-adjacent in the graph G. Hence, $|E\left(G\right)|+|E\left(\bar{G}\right)|=\frac{1}{2}\;n\left(n-1\right)$, and $V\left(G\right)=V\left(\bar{G}\right)$. Furthermore, let $J^{\left(n\right)}$ and $I_{n}$ denote the *n*-times-*n* all-ones and identity matrices, respectively. Then, by definition, it follows that the adjacency matrices of the graph G and its complement $\bar{G}$ are related by the equality

(2) $$\begin{array}{c} A\left(\bar{G}\right)=J^{\left(n\right)}-I_{n}-A\left(G\right). \end{array}$$

Let G be a *d*-regular graph on *n* vertices, and let $\bar{G}$ be the complement graph. Then, by (2), the spectra of G and $\bar{G}$ are related as follows (see Section 1.3.2 of [39]):

(3) $$\begin{array}{ccc} & & \lambda_{1}\left(\bar{G}\right)=n-d-1=n-1-\lambda_{1}\left(G\right), \end{array}$$

(4) $$\begin{array}{ccc} & & \lambda_{\ell }\left(\bar{G}\right)=-1-\lambda_{n+2-\ell }\left(G\right),\;\ell =2,\ldots ,n. \end{array}$$

Specifically, setting ℓ=n in (4) gives

(5) $$\begin{array}{c} \lambda_{\min}\left(\bar{G}\right)=-1-\lambda_{2}\left(G\right). \end{array}$$

A graph is called *acyclic* if it has no cycles, and a connected acyclic graph is called a *tree*. A tree with *n* vertices has n−1 edges, and for every pair of distinct vertices in a tree, there is a unique path joining them (see, e.g., Theorem 5.1.2 of [40]). A *leaf* in a tree is a vertex of degree 1, and every tree contains at least two leaves (a tree is therefore not a regular graph, unless it is the complete graph on two vertices $K_{2}$). A disjoint union of trees is a *forest* (i.e., a forest is an acyclic graph whose connected components are trees). In particular, a deletion of an edge from a tree gives a forest of two trees. The interested reader is referred to, e.g., Chapter 5 of [40] for further properties and analysis related to trees and forests.

Two simple graphs G=(V(G),E(G)) and H=(V(H),E(H)) are said to be *isomorphic* if there is a bijection (i.e., a one-to-one and onto mapping) f:V(G)→V(H) that preserves adjacency and non-adjacency, i.e., {u,v}∈E(G) if and only if {f(u),f(v)}∈E(H). The notation G≅H denotes that G and H are isomorphic graphs. An isomorphism from a graph to itself is called an *automorphism* of the graph.

A graph is called *self-complementary* if G and $\bar{G}$ are isomorphic graphs. These include, for example, the trivial complete graph on one vertex $K_{1}$, the length-3 path $P_{4}$, and the 5-cycle graph $C_{5}$. If G is a self-complementary graph of order *n*, then the size of G is equal to |E(G)|=12n2=14n(n−1). Since only *n* or n−1 can be even, either n≡0(mod4) or n≡1(mod4). For every such *n*, there exists a recursive algorithm for constructing a self-complementary graph of order *n* (see Exercise 29 of [44]). More explicitly, if G is a self-complementary graph of order *n*, then its disjoint union with the length-3 path $P_{4}=\left[v_{1},v_{2},v_{3},v_{4}\right]$, where each of the vertices $v_{2}$ and $v_{3}$ in $P_{4}$ is connected to all the vertices in G, gives a self-complementary graph of order n+4. Starting with a graph G that is equal to $P_{4}$ or $C_{5}$ (for graph orders of n=4 or n=5, respectively) gives, by the above suggested recursive construction, a self-complementary graph of order *n* for all integers n>1 such that n≡0(mod4) or n≡1(mod4), respectively.

A graph G is *vertex-transitive* if for every two vertices of G, there is an automorphism of G that maps one vertex to the other. Similarly, G is said to be an *edge-transitive* graph if for every two edges $\left\{u_{1},v_{1}\right\}$ and $\left\{u_{2},v_{2}\right\}$ of G, there is an automorphism f:V(G)→V(G) of the graph G, such that $\left\{u_{2},v_{2}\right\}=\left\{f\left(u_{1}\right),f\left(v_{1}\right)\right\}$. A vertex-transitive graph is necessarily regular, but the opposite does not hold in general. Unlike a vertex-transitive graph, an edge-transitive graph is not necessarily regular. Types of graph transitivity are studied, e.g., in Chapters 3–4 of [45].

All the eigenvalues of a *d*-regular graph G are bounded in absolute value by *d*, and (as mentioned above) the largest eigenvalue is equal to *d* with an eigenvector that is equal to the all-ones column vector (see, e.g., Proposition 12.1.1 of [40]). The multiplicity of the largest eigenvalue of a *d*-regular graph is 1 if and only if G is connected (see, e.g., Theorem 4.5.2 and Proposition 12.1.1 of [40]). A graph G has its eigenvalues symmetric around zero (including their multiplicities) if and only if G is bipartite (see, e.g., Theorem 4.3.2 of [40]). Hence, the smallest eigenvalue of a *d*-regular bipartite graph is equal to −d. Moreover, if G is *d*-regular and connected, then G is bipartite if and only if −d is an eigenvalue of its adjacency matrix (see, e.g., Proposition 12.1.1 of [40]). For a *d*-regular graph G, let

(6) $$\begin{array}{c} \lambda \left(G\right)≜\max_{\ell :\;\lambda_{\ell }\left(G\right)\neq \pm d}\;\left|\lambda_{\ell }\left(G\right)\right|. \end{array}$$

A *d*-regular graph G is called *Ramanujan* if

(7) $$\begin{array}{c} \lambda \left(G\right)\leq 2\sqrt{d-1}. \end{array}$$

The reason for the expression in the right-hand side of (7) is related to the Alon–Boppana bound, which addresses the question of how small can the second-largest eigenvalue be for a connected *d*-regular graph or for a sequence of connected *d*-regular graphs whose number of vertices tends to infinity (the value of the parameter *d* is kept fixed). It states that for every *d*-regular graph G on *n* vertices, with d≥3,

(8) $$\begin{array}{c} \lambda_{2}\left(G\right)\geq 2\sqrt{d-1}-O\left({\left(\log_{d}n\right)}^{-1}\right). \end{array}$$

A non-asymptotic version of (8) appears in [46] (see also Theorem 12.2.1 of [40]). The Alon–Boppana bound was first mentioned in page 95 of [47], and it was analyzed, e.g., in [46,48,49,50,51,52,53,54,55,56,57]. Moreover, all eigenvalues of a tree, with maximum degree d≥2, are (in absolute value) at most $2\sqrt{d-1}$ (see Theorem 1 of [58]). Examples of Ramanujan graphs include:

(a) The complete *d*-regular graph $K_{d+1}$, with d≥2, whose eigenvalues are equal to *d* with multiplicity 1, and −1 with multiplicity *d*;
(b) The complete bipartite graph $K_{d,d}$, with d≥2, is a *d*-regular graph whose two nonzero eigenvalues are ±d (each of multiplicity 1), and its other 2d−2 eigenvalues are zeros.
(c) The Petersen graph, which is isomorphic to the Kneser graph K(5,2), is a Ramanujan graph since it is 3-regular with the distinct eigenvalues 3, −1, and −2.

The interested reader is referred to [58] for a recent survey paper on Ramanujan graphs, and to references therein (see, e.g., [59] that proves the existence of infinite families of bipartite Ramanujan graphs of every degree greater than 2, followed by the extension of that result to bi-regular bipartite graphs; the proof in [59] uses an original technique for controlling the eigenvalues of some random matrices).

Let G be a *d*-regular graph of order *n*. The graph G is said to be a *strongly regular* graph if there exist nonnegative integers λ and μ such that the following conditions hold:

- Every pair of adjacent vertices have exactly λ common neighbors;
- Every pair of distinct and non-adjacent vertices have exactly μ common neighbors.

Such a strongly regular graph is denoted by srg(n,d,λ,μ). Some basic properties of strongly regular graphs are next introduced, which also serve in our analysis.

(a) The complement of a strongly regular graph is also strongly regular. More explicitly, the complement of srg(n,d,λ,μ) is srg(n,n−d−1,n−2d+μ−2,n−2d+λ).
(b) The four parameters of a strongly regular graph srg(n,d,λ,μ) satisfy the relation
  (9) $$\begin{array}{c} (n-d-1)\;\mu =d\;(d-\lambda -1). \end{array}$$
(c) A strongly regular graph srg(n,d,λ,μ) has at most three distinct eigenvalues. If it is connected, then $\lambda_{1}\left(G\right)=d$ (multiplicity 1), and the other two distinct eigenvalues are
  (10) $$\begin{array}{c} p_{1,2}=\frac{1}{2}\;\left[\lambda -\mu \pm \sqrt{{\left(\lambda -\mu \right)}^{2}+4\left(d-\mu \right)}\;\right], \end{array}$$
  whose respective multiplicities are given by
  (11) $$\begin{array}{c} m_{1,2}=\frac{1}{2}\left[n-1\mp \frac{2d+(n-1)(\lambda -\mu )}{\sqrt{{\left(\lambda -\mu \right)}^{2}+4\left(d-\mu \right)}}\right]. \end{array}$$
  Since multiplicities of eigenvalues must be nonnegative integers, their expressions in (11) provide further constraints on the values of n,d,λ and μ (in addition to (9)).
(d) A connected regular graph with exactly three distinct eigenvalues is strongly regular.
(e) A strongly regular graph srg(n,d,λ,μ), with μ>0, is a connected graph whose diameter is equal to 2. This holds since two non-adjacent vertices have μ>0 common neighbors, so the distance between any pair of non-adjacent vertices is equal to 2. This can be also explained by spectral graph theory since the diameter of a connected graph is strictly smaller than the number of its distinct eigenvalues (see Theorem 4.4.1 of [40]). In light of that, the above claim about the diameter holds for all graphs that are connected and strongly regular since these graphs only have three distinct eigenvalues.
(f) If μ=0, the strongly regular graph is disconnected, and it is a disjoint union of equal-sized complete graphs (i.e., a disjoint union of cliques of the same size). A disjoint union of an arbitrary number ℓ≥2 of equal-sized complete graphs, $K_{d+1}$, has the parameters srg((d+1)ℓ,d,d−1,0). In that case, $d=p_{1}$ (see (10)), so the largest and second-largest eigenvalues coincide (by (11), that common eigenvalue has multiplicity $m_{1}+1=\ell$ in the graph spectrum). A strongly regular graph G is called *primitive* if both G and its complement $\bar{G}$ are connected graphs. Otherwise, G is called *imprimitive*. An imprimitive graph is, therefore, either a disjoint union of equal-sized complete graphs or its complement, which is a non-empty complete multipartite graph. A strongly regular graph G is imprimitive if and only if 0 or −1 is an eigenvalue of G.
(g) Let G be a primitive strongly regular graph srg(n,d,λ,μ) with the largest eigenvalue *d* (multiplicity 1), second-largest eigenvalue $r=p_{1}$ (multiplicity $m_{1}$), and smallest eigenvalue $s=p_{2}$ (multiplicity $m_{2}$). By (3) and (4), the complement $\bar{G}$ is a primitive strongly regular graph, having the largest eigenvalue n−d−1 (multiplicity 1), second-largest eigenvalue −1−s (multiplicity $m_{2}$), and smallest eigenvalue −1−r (multiplicity $m_{1}$). Each of these primitive strongly regular graphs has three distinct eigenvalues.

The interested reader is referred to [60], which is focused on the properties and constructions of strongly regular graphs.

Part of this work is focused on the strong product of graphs, which is next defined.

**Definition** **1.** *Let $G_{1}$ and $G_{2}$ be two graphs. The strong product $G=G_{1}⊠G_{2}$ is a graph whose vertex set is $V\left(G\right)=V\left(G_{1}\right)\times V\left(G_{2}\right)$ (i.e., a Cartesian product of the two vertex sets), and distinct vertices $\left\{u_{1},u_{2}\right\}$ and $\left\{v_{1},v_{2}\right\}$ in G are adjacent if one of the following three conditions is satisfied:*

*(a)*  *$u_{1}=v_{1}$ and $\left\{u_{2},v_{2}\right\}\in E\left(G_{2}\right)$,*
*(b)*  *$\left\{u_{1},v_{1}\right\}\in E\left(G_{1}\right)$ and $u_{2}=v_{2}$,*
*(c)*  *$\left\{u_{1},v_{1}\right\}\in E\left(G_{1}\right)$ and $\left\{u_{2},v_{2}\right\}\in E\left(G_{2}\right)$.*

A strong product of graphs is commutative in the sense that

(12) $$\begin{array}{c} G_{1}⊠G_{2}≅G_{2}⊠G_{1}, \end{array}$$

and it is also associative in the sense that

(13) $$\begin{array}{c} \left(G_{1}⊠G_{2}\right)⊠G_{3}≅G_{1}⊠\left(G_{2}⊠G_{3}\right), \end{array}$$

for every three graphs $G_{1},G_{2}$ and $G_{3}$ (see Proposition 4.1 of [11]).

This paper relies on the *Lovász θ-function* of a graph [6], to be next introduced.

**Definition** **2.** *Let G be a simple graph. An orthogonal representation of G in the d-dimensional Euclidean space $\left(R^{d}\right)$ assigns to each vertex i∈V(G) a vector $u_{i}\in R^{d}$ such that $u_{i}^{T}u_{j}=0$ if {i,j}∉E(G). In other words, the vertices of a simple graph are assigned vectors in $R^{d}$ such that the vectors that are assigned to any pair of distinct and non-adjacent vertices of that graph are orthogonal. An orthonormal representation of G is an orthogonal representation of that graph such that all representing vectors have unit length.*

**Remark** **1.** *In an orthogonal representation of a graph G, non-adjacent vertices are mapped to orthogonal vectors, although adjacent vertices are not necessarily mapped to non-orthogonal vectors. If the latter condition is satisfied, then the orthogonal representation is said to be faithful.*

**Definition** **3.** *Let G be a finite, undirected and simple graph. Its* Lovász θ-function *is defined as*

(14) $$\begin{array}{c} \theta \left(G\right)≜\min_{u,c}\;\max_{i\in V(G)}\;\frac{1}{{\left(c^{T}u_{i}\right)}^{2}}\;, \end{array}$$

*where the minimum is taken over all orthonormal representations $\left\{u_{i}:i\in V\left(G\right)\right\}$ of G, and all unit vectors c. The unit vector c is called the handle of the orthonormal representation. (By the Cauchy-Schwarz inequality, $|c^{T}u_{i}|\leq ∥c∥\;∥u_{i}∥=1$, so θ(G)≥1 with equality if and only if G is a complete graph).*

The Lovász θ-function of a graph can be written as a semidefinite program, which satisfies strong duality ([5,61,62], and Section 11.2 of [63]). This enables to compute the value of θ(G) in polynomial time [61]. More precisely, there is an algorithm that computes, for every graph G and every ε>0, a real number *t* such that |θ(G)−t|<ε, where the running time of the algorithm is polynomial in n≜|V(G)| and $\log\left(\frac{1}{\varepsilon }\right)$ (see Theorem 11.11 of [63]). The following properties of the Lovász θ-function are used throughout this paper:

(a) The sandwich theorem ([5,61], Lemma 3.2.4 of [62], and Theorem 11.1 of [63]) is stated in the two equivalent forms
  (15) $$\begin{array}{ccc} & & \alpha \left(G\right)\leq \theta \left(G\right)\leq \chi \left(\bar{G}\right), \end{array}$$
  (16) $$\begin{array}{ccc} & & \omega \left(G\right)\leq \theta \left(\bar{G}\right)\leq \chi \left(G\right). \end{array}$$
(b) Theorem 7 of [6]: The Lovász θ-function factorizes for the strong product of graphs, i.e.,
  (17) $$\begin{array}{c} \theta \left(G_{1}⊠G_{2}\right)=\theta \left(G_{1}\right)\;\theta \left(G_{2}\right). \end{array}$$
(c) Corollary 2 and Theorem 8 of [6]:
  (18) $$\begin{array}{c} \theta \left(G\right)\;\theta \left(\bar{G}\right)\geq \left|V\left(G\right)\right|, \end{array}$$
  with equality in (18) if the graph G is vertex-transitive.
(d) Theorem 9 of [6]: Let G be a *d*-regular graph of order *n*. Then,
  (19) $$\begin{array}{c} \theta \left(G\right)\leq -\frac{n\;\lambda_{n}\left(G\right)}{d-\lambda_{n}\left(G\right)}, \end{array}$$
  where $\lambda_{n}\left(G\right)<0$, unless G is an empty graph. Equality holds in (19) if G is edge-transitive.
(e) Two simple observations relating the Lovász θ-functions of a graph and its subgraphs:
  - If F is a spanning subgraph of a graph G, then θ(F)≥θ(G).
  - If F is an induced subgraph of a graph G, then θ(F)≤θ(G).
(f) Theorem 2 of [14]: Although unrelated to the analysis in this paper, another interesting property of the Lovász θ-function is given by the identity
  (20) $$\begin{array}{c} \underset{H}{\sup}\frac{\alpha (G⊠H)}{\theta (G⊠H)}=1, \end{array}$$
  which holds for every simple, finite, and undirected graph G, and where the supremum in the left-hand side of (20) is taken over all such graphs H. This shows that the leftmost inequality in (15) can be made arbitrarily tight by looking at the strong product of the given graph G with a suitable graph H.

The Shannon capacity of a simple, finite, and undirected graph G was introduced in [1] to determine the maximum information rate that enables error-free communication. To that end, a discrete memoryless communication channel is represented by a *confusion graph* G that is constructed as follows. The vertices in the graph are represented by the symbols of the input alphabet to that channel, and any two distinct vertices in that graph are adjacent if the corresponding two input symbols are not distinguishable by the channel (in the sense that there exists an output symbol such that the transition probabilities from each of these two input symbols to that output symbol are strictly positive). This means that the exact values of the positive transition probabilities of the channel, as well as the output alphabet of the channel, are irrelevant to the construction of the confusion graph G. The rationality in doing so is the interest to pictorially represent (by a graph) all those pairs of input symbols that are not distinguishable by the channel. Consider a transmission of *k*-length strings. The *k-th confusion graph* of the channel is defined as

(21) $$\begin{array}{c} G^{⊠\;k}≜\underset{k-1\;\mathrm{strong}\;\mathrm{products}}{\underset{︸}{G⊠\ldots ⊠G}}, \end{array}$$

which is the *k*-fold strong power of G. This is because the independence number of $G^{⊠\;k}$ is equal to the maximum number of *k*-length strings at the channel input that can be transmitted with error-free communication (indeed, a pair of non-adjacent vertices in $G^{⊠\;k}$ represent *k*-length strings that can be distinguished by the channel, as a result of having a common position in these two input strings where the corresponding two symbols at that position are distinguishable by the channel). Consequently, the maximum *information rate per symbol* that is achievable by using input strings of length *k* is equal to $\frac{1}{k}\;\log\alpha \left(G^{⊠\;k}\right)=\log\sqrt[{k}]{\alpha (G^{⊠\;k})}$, for all k∈N (i.e., it is the logarithm of the maximum number of *k*-length input strings that are distinguishable by the channel, normalized by the length *k*). The *Shannon capacity of a graph* G is defined to be the (exponent of the) maximum information rate per symbol that is achievable with error-free communication, where the transmission takes place over a discrete memoryless channel whose confusion graph is equal to G, and the length of the input strings to the channel is unlimited. It is denoted by Θ(G) (recall that the Lovász θ-function is denoted by θ(G)). Taking the supremum over *k*, the Shannon capacity of G is given by (see [1], and Chapter 42 of [64])

(22) $$\begin{array}{c} \Theta \left(G\right)=\underset{k\in N}{\sup}\sqrt[{k}]{\alpha (G^{⊠\;k})}. \end{array}$$

The Shannon capacity can be rarely computed exactly (see, e.g., [1,3,6,7,8,9,10,29]). Analytical observations that also explain why it is, in general, even difficult to approximate it are addressed in [15,26]. Calculable upper bounds on Θ(G) were derived by Shannon [1], Lovász [6], Haemers [7], and more recently by Hu et al. [29]. The Lovász θ-function θ(G) is a calculable upper bound on the graph capacity Θ(G), i.e.,

(23) $$\begin{array}{c} \alpha (G)\leq \Theta (G)\leq \theta (G), \end{array}$$

where the leftmost inequality in (23) follows from (22) (by setting k=1), and the rightmost inequality in (23) is Theorem 1 of [6]. In regard to the rightmost inequality in (23), it is also shown in [8] that the Lovász θ-function of a graph, θ(G), cannot be upper bounded by any function of its Shannon capacity Θ(G). As mentioned above, the computational task of the Lovász θ-function, θ(G), is in general feasible by semidefinite programming. Fundamental graph parameters such as its Shannon capacity Θ(G), independence number α(G), clique number ω(G), and chromatic number χ(G) are all NP-hard problems. The polynomial-time computability of the Lovász θ-function of a graph makes inequalities (15), (16), and (23) very useful in obtaining polynomial-time computable upper bounds on the independence number, clique number, and the Shannon capacity of a graph, as well as having such a computable lower bound on the chromatic number.

## 3. Theorems, Discussions and Examples

The present section provides the results of this work, followed by examples and discussions. It is composed of five subsections that address issues related to the Lovász θ-function, Shannon capacity of graphs, Ramanujan graphs, eigenvalues, and chromatic numbers of strong graph products or strong graph powers.

### 3.1. Bounds on Lovász θ-Function, and an Exact Result for Strongly Regular Graphs

Let G be a *d*-regular graph with *n* vertices, and let $\bar{G}$ be the complement graph that is an (n−d−1)-regular graph of order *n*. An upper bound on θ(G) and a lower bound on $\theta (\bar{G})$ were obtained by Lovász, expressed in terms of the smallest eigenvalue of the adjacency matrix of G (see Theorem 9 and Corollary 3 of [6]). The novelties in the next result (Proposition 1) are as follows:

(a) It forms a counterpart of a bound by Lovász (Theorem 9 of [6]), providing a lower bound on θ(G) and an upper bound on $\theta (\bar{G})$ that are both expressed in terms of the second-largest eigenvalue of the adjacency matrix of G.
(b) It asserts that these two pairs of upper and lower bounds on θ(G) and $\theta (\bar{G})$ are tight for the family of strongly regular graphs. This gives a simple closed-form expression of the Lovász θ-function of a strongly regular graph srg(n,d,λ,μ) (and the complement graph) as a function of its four parameters.
(c) Further sufficient conditions for the tightness of these bounds are provided.

**Proposition** **1.** *Let G be a d-regular graph of order n, which is a non-complete and non-empty graph. Then, the following bounds hold for the Lovász θ-function of G and its complement $\bar{G}$:*

*(a)* 
(24) $$\begin{array}{c} \frac{n-d+\lambda_{2}\left(G\right)}{1+\lambda_{2}\left(G\right)}\leq \theta \left(G\right)\leq -\frac{n\lambda_{n}\left(G\right)}{d-\lambda_{n}\left(G\right)}. \end{array}$$
  - *Equality holds in the leftmost inequality of (24) if $\bar{G}$ is both vertex-transitive and edge-transitive, or if G is a strongly regular graph;*
  - *Equality holds in the rightmost inequality of (24) if G is edge-transitive, or if G is a strongly regular graph.*
*(b)* 
(25) $$\begin{array}{c} 1-\frac{d}{\lambda_{n}\left(G\right)}\leq \theta \left(\bar{G}\right)\leq \frac{n\left(1+\lambda_{2}\left(G\right)\right)}{n-d+\lambda_{2}\left(G\right)}. \end{array}$$
  - *Equality holds in the leftmost inequality of (25) if G is both vertex-transitive and edge-transitive, or if G is a strongly regular graph;*
  - *Equality holds in the rightmost inequality of (25) if $\bar{G}$ is edge-transitive, or if G is a strongly regular graph.*

**Proof.**  See Section 4.1.1. □

**Remark** **2.** *In light of the sufficient conditions for each of the four inequalities in Proposition 1 to hold with equality, define the following subfamilies of regular graphs:*

- *Let $G_{1}$ be the family of graphs G such that $\bar{G}$ is both vertex-transitive and edge-transitive;*
- *Let $G_{2}$ be the family of regular and edge-transitive graphs;*
- *Let $G_{3}$ be the family of graphs G such that $\bar{G}$ is regular and edge-transitive;*
- *Let $G_{4}$ be the family of graphs that are both vertex-transitive and edge-transitive;*
- *Let $G_{5}$ be the family of the strongly regular graphs.*

*We next show by explicit examples, obtained by using the SageMath software [65], that none of the families $G_{1}$, $G_{2}$ $G_{3}$ and $G_{4}$ is included in the family $G_{5}$, and vice versa.*

*(a)*  *The Cameron graph is a strongly regular graph srg(231,30,9,3) (see Section 10.54 of [60]). Its complement is vertex-transitive (hence, regular), but not edge-transitive. This shows that $G_{5}⊈G_{3}$, so also $G_{5}⊈G_{1}$.*
*(b)*  *The complement of the Cameron graph is a strongly regular graph srg(231,200,172,180); it is vertex-transitive (hence, regular), but not edge-transitive. This shows that $G_{5}⊈G_{2}$, so also $G_{5}⊈G_{4}$.*
*(c)*  *The Foster graph is 3-regular on 90 vertices (see page 305 of [60]), which is vertex-transitive and edge-transitive, but it is not strongly regular. This shows that $G_{4}⊈G_{5}$, so also $G_{2}⊈G_{5}$.*
*(d)*  *The complement of the Foster graph is an 86-regular graph on 90 vertices, whose complement (i.e., the Foster graph) is vertex-transitive and edge-transitive, but it is not strongly regular. This shows that $G_{1}⊈G_{5}$, so also $G_{3}⊈G_{5}$.*

The next result provides a closed-form expression of the Lovász θ-function for strongly regular graphs (and their strongly regular complements). This result relies on Proposition 1 and the closed-form expressions of the distinct eigenvalues of a strongly regular graph.

**Corollary** **1.** *Let G be a strongly regular graph with parameters srg(n,d,λ,μ). Then,*

(26) $$\begin{array}{c} \theta \left(G\right)=\frac{n\;(t+\mu -\lambda )}{2d+t+\mu -\lambda }, \end{array}$$

*and*

(27) $$\begin{array}{cc} \theta (\bar{G}) & =\frac{n}{\theta (G)} \end{array}$$

(28) $$\begin{array}{cc} \; & =1+\frac{2d}{t+\mu -\lambda }, \end{array}$$

*where*

(29) $$\begin{array}{c} t≜\sqrt{{\left(\mu -\lambda \right)}^{2}+4\left(d-\mu \right)}. \end{array}$$

*Furthermore, if 2d+(n−1)(λ−μ)≠0, then θ(G) and $\theta (\bar{G})$ are rational numbers.*

**Proof.**  See Section 4.1.2. □

**Remark** **3.** *By (27), if G is a strongly regular graph on n vertices, then $\theta \left(G\right)\;\theta \left(\bar{G}\right)=n$. This relation is also known to hold if the graph G is vertex-transitive (see Theorem 8 of [6]). It should be noted that not all the strongly regular graphs are necessarily vertex-transitive, so the observation here is not implied by Theorem 8 of [6]. As a counter example for strongly regular graphs that are not vertex-transitive, consider the Chang graphs. These are three of the existing four non-isomorphic strongly regular graphs with parameters srg(28,12,6,4) (see Section 10.11 of [60]); the fourth such graph, denoted by $T_{8}$, is the line graph of the complete graph on 8 vertices $K_{8}$. The three Chang graphs are not vertex-transitive and also not edge-transitive (in contrast to $T_{8}$ that is vertex-transitive and edge-transitive), as it can be verified by the SageMath software [65].*

**Remark** **4.** *The 5-cycle $C_{5}$ is a strongly regular graph srg(5,2,0,1). Its Lovász θ-function coincides with its Shannon capacity, being equal to $\sqrt{5}$ (see Theorem 2 of [6]). Although it is an irrational number, it is consistent with Corollary 1 since 2d+(n−1)(λ−μ)=2·2+4(0−1)=0.*

### 3.2. Eigenvalue Inequalities, Strongly Regular Graphs, and Ramanujan Graphs

The present subsection relies on Proposition 1, with the following contributions:

(a) Derivation of inequalities that relate the second-largest and smallest eigenvalues of a regular graph. These inequalities are proved to hold with equality if and only if the graph is strongly regular.
(b) Derivation of bounds on parameters of Ramanujan graphs.
(c) A more general result is presented for a sequence of regular graphs whose degrees scale sub-linearly with the orders of these graphs, and their orders tend to infinity.

This subsection is composed of Corollaries 2–6, which all rely on Proposition 1. It starts by providing eigenvalue inequalities.

**Corollary** **2.** *Let G be a d-regular graph of order n, which is non-complete and non-empty. Then,*

(30) $$\begin{array}{c} \lambda_{n}\left(G\right)\leq -\frac{d\;(n-d+\lambda_{2}\left(G\right))}{d+\left(n-1\right)\;\lambda_{2}\left(G\right)}, \end{array}$$

*or equivalently,*

(31) $$\begin{array}{c} \lambda_{2}\left(G\right)\geq -\frac{d\;(n-d+\lambda_{n}\left(G\right))}{d+\left(n-1\right)\;\lambda_{n}\left(G\right)}. \end{array}$$

*Furthermore, inequalities (30) and (31) hold with equality if and only if G is a strongly regular graph.*

**Proof.**  See Section 4.2.1. □

The next result introduces, in part, Nordhaus–Gaddum type inequalities for the second-largest and smallest eigenvalues of regular graphs, which are tight for all strongly regular graphs. Regarding Nordhaus–Gaddum type inequalities, the interested reader is referred to [66,67,68].

**Corollary** **3.** *Let G be a d-regular graph of order n, which is non-complete and non-empty, and let*

(32) $$\begin{array}{c} g_{\ell }\left(G\right)≜\lambda_{\ell }\left(\bar{G}\right)-\frac{d(n-d+\lambda_{\ell }\left(G\right))}{d+\left(n-1\right)\lambda_{\ell }\left(G\right)},\;\forall \;\ell \in \left[n\right]. \end{array}$$

*The following holds:*

*(a)* 
(33) $$\begin{array}{c} g_{n}\left(G\right)\leq -1\leq g_{2}\left(G\right), \end{array}$$
  *and the two inequalities in (33) hold with equality if and only if G is strongly regular.*
*(b)*  *If G is a strongly regular graph, then the number of distinct values in the sequence ${\left\{g_{\ell }\left(G\right)\right\}}_{\ell =1}^{n}$ is either 2 or 3, and*
  - *it is equal to 2 if the multiplicities of the second-largest and smallest eigenvalues of G are identical in the subsequence $\left(\lambda_{2}\left(G\right),\ldots ,\lambda_{n}\left(G\right)\right)$;*
  - *it is otherwise equal to 3.*
*(c)*  *If G is self-complementary, then*
(34) $$\begin{array}{ccc} & & \lambda_{2}\left(G\right)\geq \frac{1}{2}\left(\sqrt{n}-1\right), \end{array}$$
(35) $$\begin{array}{ccc} & & \lambda_{n}\left(G\right)\leq -\frac{1}{2}\left(\sqrt{n}+1\right). \end{array}$$
*(d)*  *If G is self-complementary and strongly regular, then (34) and (35) hold with equality.*

**Proof.**  See Section 4.2.2. □

**Example** **1.** *Item (b) of Corollary 3 refers to the dichotomy in the number of distinct values in the sequence ${\left\{g_{\ell }\left(G\right)\right\}}_{\ell =1}^{n}$. This statement applies to all strongly regular graphs (either connected or disconnected). We believe that the following example contributes to its clarity, in addition to its formal proof in Section 4.2.2. Let G be a disjoint union of the three complete graphs $K_{2}$, which gives a disconnected strongly regular graph. Its complement is the complete 3-partite graph $\bar{G}=K_{2,2,2}$, so n=6, and*

(36) $$\begin{array}{ccc} & & {\left\{\lambda_{\ell }\left(G\right)\right\}}_{\ell =1}^{6}=\left(1,\;1,\;1,-1,-1,-1\right), \end{array}$$

(37) $$\begin{array}{ccc} & & {\left\{\lambda_{\ell }\left(\bar{G}\right)\right\}}_{\ell =1}^{6}=\left(4,\;0,\;0,\;0,-2,-2\right), \end{array}$$

(38) $$\begin{array}{ccc} & & {\left\{g_{\ell }\left(G\right)\right\}}_{\ell =1}^{6}=\left(3,-1,-1,\;1,-1,-1\right). \end{array}$$

*By (36), the multiplicities of $\lambda_{2}\left(G\right)$ and $\lambda_{n}\left(G\right)$ in the spectrum of G are identical, but these multiplicities are distinct in the subsequence ${\left\{\lambda_{\ell }\left(G\right)\right\}}_{\ell =2}^{6}=\left(1,\;1,-1,-1,-1\right)$. Hence, the fact that the sequence ${\left\{g_{\ell }\left(G\right)\right\}}_{\ell =1}^{6}$ gets three distinct values is indeed consistent with the claim in Item (b) of Corollary 3.*

**Example** **2.** *Let G be the Hall-Janko graph, which is a strongly regular graph with parameters srg(100,36,14,12) (see Section 10.32 of [60]). As a numerical verification of Item (b) in Corollary 3 (and its proof in Section 4.2.2), the sequence ${\left\{g_{\ell }\left(G\right)\right\}}_{\ell =1}^{100}$ in (32) gets the three distinct values: n−d−2=62 (at ℓ=1), −1 (for 2≤ℓ≤37 or 65≤ℓ≤100), and 9 (for 38≤ℓ≤64). The third value (9) is attained by the sequence $\left\{g_{\ell }\left(G\right)\right\}$ twenty-seven times. By the proof of Item (b) in Corollary 3, the multiplicity of the third value (27) is equal to the absolute value of the difference between the multiplicities of the second-largest and smallest eigenvalues of the graph G. The spectrum of the graph G is given by $36^{1}6^{36}{\left(-4\right)}^{63}$ (this can be verified by (10) and (11)), and the above difference (in absolute value) is indeed equal to |63−36|=27. Next, let G be the 5-cycle graph $C_{5}$, which is a strongly regular graph with parameters srg(5,2,0,1). The second-largest and smallest eigenvalues of G are equal to $\frac{1}{2}\left(\sqrt{5}-1\right)$ and $-\frac{1}{2}\left(\sqrt{5}+1\right)$, respectively, and their multiplicities coincide, being both equal to 2. In light of Item (b) in Corollary 3, the sequence ${\left\{g_{\ell }\left(G\right)\right\}}_{\ell =1}^{5}$ in (32) gets only two distinct values: n−d−2=1 at ℓ=1, and −1 for 2≤ℓ≤5.*

**Remark** **5.** *We discuss here an implication of the conditions for equalities in Proposition 1. Let G be a d-regular graph. Inequality (30) holds with equality if and only if both inequalities in (24) hold with equality. By Item (a) of Proposition 1, the leftmost inequality in (30) holds with equality if $\bar{G}$ is both vertex-transitive and edge-transitive, and the rightmost inequality in (30) holds with equality if G is edge-transitive. Combining both sufficient conditions for the two inequalities in (30) to hold with equality, it follows that a sufficient condition for equality in (30) is given by the requirement that G and $\bar{G}$ are both vertex-transitive and edge-transitive (recall that $\bar{G}$ is vertex-transitive if and only if G is so).*

*By Corollary 2, inequality (30) holds with equality if and only if G is strongly regular. By a comparison of the former (sufficient) condition with the latter (necessary and sufficient) condition for equality to hold in (30), it follows that if G and $\bar{G}$ are both vertex-transitive and edge-transitive, then G is strongly regular.*

*It should be noted, with gratitude to an anonymous reviewer, that a stronger result can be obtained by replacing the requirement of vertex-transitivity with the weaker condition of regularity. Namely, if G is regular, and G and $\bar{G}$ are both edge-transitive, then G is strongly regular. This can be shown as follows.*

- *(G is edge transitive) ⇒ (every edge in G is contained in the same number of triangles) ⇔ (every pair of adjacent vertices in G has the same number of common neighbors);*
- *($\bar{G}$ is edge transitive) ⇒ (for every edge $\left\{u,v\right\}\in E\left(\bar{G}\right)$, the same number of vertices are not adjacent in $\bar{G}$ to either u or v) ⇔ (every pair of non-adjacent vertices in G has the same number of common neighbors);*
- *G is regular (by assumption);*

*and these three observations correspond to the conditions in the definition of a strongly regular graph.*

**Example** **3.** *The Schläfli graph $G_{1}$ is 16-regular on 27 vertices. Both $G_{1}$ and its complement are edge-transitive. By Remark 5, the graph $G_{1}$ is indeed strongly regular; its parameters are given by srg(27,16,10,8) (see Section 10.10 of [60]).*

*Consider, on the other hand, the Shrikhande graph $G_{2}$ which is 6-regular on 16 vertices. It is a strongly regular graph with parameters srg(16,6,2,2) (see Section 10.6 of [60]). The graph $G_{2}$ is edge-transitive, but its complement is not edge-transitive (this was verified by the SageMath software [65]). In addition, the Cameron graph $G_{3}$ is a strongly regular graph srg(231,30,9,3) (see Section 10.54 of [60]). It can be verified that it is edge-transitive, and that its complement is not edge-transitive. This shows that the family of regular graphs G with the property that G and its complement $\bar{G}$ are both edge-transitive is a strict subset of the family of strongly regular graphs.*

**Remark** **6.** *In continuation to Example 3, strongly-regular graphs G such that G and $\bar{G}$ are both edge-transitive include, e.g., the Hall-Janko, Hoffman-Singleton, Mesner, Petersen, Schläfli, Sims-Gewirtz, and Suzuki graphs (this has been verified by the SageMath software [65]; for the introduction of these graphs, the reader is referred to [60]). It also includes the infinite families of Paley and Peisert graphs [69,70], which are self-complementary and arc-transitive graphs. (All self-complementary and arc-transitive graphs are strongly regular).*

**Remark** **7.** *In connection to Remark 5, two related statements have been proved by Neumaier:*

(a) *A connected, edge-transitive and strongly regular graph is vertex-transitive (Lemma 1.3 of [71]).*
(b) *A vertex-transitive and edge-transitive graph containing a regular clique is strongly regular (Corollary 2.4 of [72]). (A clique C is called regular if every vertex not in C is adjacent to the same positive number of vertices in C).*

*Strongly regular graphs that are both vertex- and edge-transitive are studied in [73].*

**Corollary** **4.** *Let ${\left\{G_{\ell }\right\}}_{\ell \in N}$ be a sequence of graphs where $G_{\ell }$ is $d_{\ell }$-regular of order $n_{\ell }$, and*

(39) $$\begin{array}{c} \lim_{\ell \to \infty }n_{\ell }=\infty ,\;\lim_{\ell \to \infty }\;\frac{d_{\ell }}{n_{\ell }}=0. \end{array}$$

*Then,*

(40) $$\begin{array}{ccc} & & \underset{\ell \to \infty }{lim sup}\;\omega \left(G_{\ell }\right)\leq a, \end{array}$$

(41) $$\begin{array}{ccc} & & \underset{\ell \to \infty }{lim inf}\;\frac{\chi ({\bar{G}}_{\ell })}{n_{\ell }}\geq \frac{1}{a}, \end{array}$$

*with*

(42) $$\begin{array}{c} a≜1+\underset{\ell \to \infty }{lim sup}\;\left\lfloor \lambda_{2}\left(G_{\ell }\right)\right\rfloor . \end{array}$$

**Proof.**  See Section 4.2.3. □

**Corollary** **5.** *Let ${\left\{G_{\ell }\right\}}_{\ell \in N}$ be a sequence of connected, Ramanujan, and d-regular graphs where d∈N is fixed, $G_{\ell }$ is a graph on $n_{\ell }$ vertices, and $\lim_{\ell \to \infty }n_{\ell }=\infty$. Then,*

(43) $$\begin{array}{ccc} & & \underset{\ell \to \infty }{lim sup}\;\omega \left(G_{\ell }\right)\leq 1+\left\lfloor 2\sqrt{d-1}\right\rfloor , \end{array}$$

(44) $$\begin{array}{ccc} & & \underset{\ell \to \infty }{lim inf}\;\frac{\theta (G_{\ell })}{n_{\ell }}\geq \frac{1}{1+2\sqrt{d-1}}, \end{array}$$

(45) $$\begin{array}{ccc} & & \underset{\ell \to \infty }{lim inf}\;\frac{\chi ({\bar{G}}_{\ell })}{n_{\ell }}\geq \frac{1}{1+\lfloor 2\sqrt{d-1}\rfloor }. \end{array}$$

**Proof.**  See Section 4.2.4. □

In continuation to Corollary 5, the following result provides non-asymptotic bounds on some graph parameters.

**Corollary** **6.** *Let G be a connected, Ramanujan, and d-regular graph with n vertices. Then,*

(46) $$\begin{array}{ccc} & & \omega \left(G\right)\leq \left\lfloor \frac{n\left(1+2\sqrt{d-1}\;\right)}{n-d+2\sqrt{d-1}}\right\rfloor \;, \end{array}$$

(47) $$\begin{array}{ccc} & & \theta \left(G\right)\geq \frac{n-d+2\sqrt{d-1}}{1+2\sqrt{d-1}}\;, \end{array}$$

(48) $$\begin{array}{ccc} & & \chi \left(\bar{G}\right)\geq \left\lceil \frac{n-d+2\sqrt{d-1}}{1+2\sqrt{d-1}}\;\right\rceil . \end{array}$$

**Proof.**  See Section 4.2.5. □

**Remark** **8.** *Inequalities (40), (43), and (46) can be also obtained from Theorem 2.1.3 of [74], which states that for an arbitrary simple, finite, and undirected graph G on n vertices, whose maximal degree is given by Δ(G), the following bound on the clique number of G holds:*

(49) $$\begin{array}{c} \omega \left(G\right)\leq \frac{n\left(d+\lambda_{1}\left(G\right)\;\lambda_{2}\left(G\right)\right)}{dn-\Delta {\left(G\right)}^{2}+\lambda_{1}\left(G\right)\;\lambda_{2}\left(G\right)}. \end{array}$$

*For a d-regular graph G on n vertices, we have $\lambda_{1}\left(G\right)=d=\Delta \left(G\right)$, which then specializes (49) to (see Theorem 2.1.4 of [74])*

(50) $$\begin{array}{c} \omega \left(G\right)\leq \frac{n\left(1+\lambda_{2}\left(G\right)\right)}{n-d+\lambda_{2}\left(G\right)}. \end{array}$$

*Inequality (46) can be also obtained from (50), combined with the satisfiability of the inequality $\left|\lambda_{2}\left(G\right)\right|\leq 2\sqrt{d-1}$ if G is a connected, d-regular, and Ramanujan graph, together with the fact that the right-hand side of (50) is monotonically increasing in the parameter $\lambda_{2}\left(G\right)$.*

### 3.3. Bounds on Eigenvalues of Strong Products of Regular Graphs

A small second-largest eigenvalue of the adjacency matrix of a regular graph implies that the graph is a good expander (see, e.g., Theorem 12.1.2 of [40], and [47,75,76,77,78,79]).

The Alon–Boppana bound in (8) is a lower bound on the second-largest eigenvalue of a regular graph (see Section 2). By that bound, for every sequence ${\left\{G_{k}\right\}}_{k=1}^{\infty }$ of *d*-regular graphs, with a fixed integer d≥3 and orders tending to infinity (i.e., $\lim_{k\to \infty }\left|V\left(G_{k}\right)\right|=\infty$), the second-largest eigenvalues of their adjacency matrices satisfy

(51) $$\begin{array}{c} \underset{k\to \infty }{lim inf}\lambda_{2}\left(G_{k}\right)\geq 2\sqrt{d-1}, \end{array}$$

(see, e.g., Theorem 12.1.2 of [40]). This lower bound is asymptotically tight, and its tightness can be strengthened beyond the second-largest eigenvalue. More explicitly, by Serre’s theorem [56], for every fixed integer d≥3 and (an arbitrarily small) ε>0, there exists a positive constant c=c(ε,d) such that every *d*-regular graph with *n* vertices has at least cn eigenvalues that are larger than or equal to $2\sqrt{d-1}-\varepsilon$. In other words, Serre’s theorem states that, for every desired accuracy, a non-vanishing fraction of the *n* eigenvalues of every *d*-regular graph has the property of satisfying the Alon–Boppana lower bound within that accuracy (see a simplified proof in Theorem 12.2.3 of [40] or Theorem 1 of [51]). Analogous theorems, concerning the least eigenvalues of *d*-regular graphs, also hold under an additional hypothesis that the graphs do not have odd cycles below a certain length (see Section 4 of [51]). It overall justifies the definition of Ramanujan graphs (see (7)) as *d*-regular graphs, with an integer d≥3, whose all non-trivial eigenvalues are (in absolute value) at most $2\sqrt{d-1}$.

For a *k*-fold strong power of a *d*-regular graph, the degree is increased exponentially in *k*, being equal to $d_{k}={\left(1+d\right)}^{k}-1$. It is therefore of interest to obtain an alternative lower bound on the second-largest eigenvalue of strong products of regular graphs. Its derivation is motivated by the significance of strong products, and in particular strong powers of a given graph:

(1) The graph capacity in Shannon’s problem of zero-error communication [1] is given in (22), which is expressed in terms of the independence numbers of all *k*-fold strong powers of the graph (with k∈N);
(2) The Witsenhausen rate [13] in the zero-error source coding problem, with perfect side information at the receiver, is expressed in a dual form to (22), where the independence numbers of *k*-fold strong powers of a graph (with k∈N) are replaced by their chromatic numbers, and the supremum over *k* is replaced by an infimum (see Section 3 of [3]);
(3) There exists a polynomial-time algorithm that finds the unique prime factorization of any connected graph under the operation of strong graph multiplication [12].

It is demonstrated in this section that, under a certain condition, the suggested lower bound on the second-largest eigenvalue of the *k*-fold strong power of a regular graph offers a larger exponential growth rate in *k*, as compared to the Alon–Boppana bound.

The following proposition provides a lower bound on the second-largest eigenvalue, and an upper bound on the smallest eigenvalue of the adjacency matrices of strong products of regular graphs. Their derivation relies on Proposition 1, jointly with the factorization property in (17). Both bounds are expressed in terms of the Lovász θ-function of each factor. This enables to obtain analytical bounds on the second-largest and smallest eigenvalues of a *k*-fold strong power of a regular graph, with a low computational complexity that is not affected by *k*. It stays in contrast to the computational complexity of these eigenvalues, which significantly increases with *k*.

**Proposition** **2.** *Let $G_{1},\ldots ,G_{k}$ be regular graphs such that, for all ℓ∈[k], the graph $G_{\ell }$ is $d_{\ell }$-regular of order $n_{\ell }$. The following bounds hold for their strong product:*

*(a)*  *Unless all $G_{\ell }$ (with ℓ∈[k]) are complete graphs, then*
(52) $$\begin{array}{cc} \lambda_{2}\left(G_{1}⊠\ldots ⊠G_{k}\right)\; & \geq \frac{\prod_{\ell =1}^{k}n_{\ell }-\prod_{\ell =1}^{k}\left(1+d_{\ell }\right)}{\prod_{\ell =1}^{k}\theta \left(G_{\ell }\right)-1}-1 \end{array}$$
(53) $$\begin{array}{cc} \; & \geq \frac{\prod_{\ell =1}^{k}n_{\ell }-\prod_{\ell =1}^{k}\left(1+d_{\ell }\right)}{\prod_{\ell =1}^{k}\left(-\frac{n_{\ell }\;\lambda_{\min}\left(G_{\ell }\right)}{d_{\ell }-\lambda_{\min}\left(G_{\ell }\right)}\right)-1}-1, \end{array}$$
  *and inequality (53) holds with equality if, for all ℓ∈[k], the regular graph $G_{\ell }$ is either edge-transitive or strongly regular.*
*(b)*  *Unless all $G_{\ell }$ (with ℓ∈[k]) are empty graphs, then*
(54) $$\begin{array}{c} \lambda_{\min}\left(G_{1}⊠\ldots ⊠G_{k}\right)\leq -\frac{\prod_{\ell =1}^{k}\left(1+d_{\ell }\right)-1}{\prod_{\ell =1}^{k}\left(\frac{n_{\ell }}{\theta (G_{\ell })}\right)-1}\;. \end{array}$$
  *If each regular graph $G_{\ell }$ (with ℓ∈[k]) is either edge-transitive or strongly regular, then (54) can be expressed in an equivalent form as*
(55) $$\begin{array}{c} \lambda_{\min}\left(G_{1}⊠\ldots ⊠G_{k}\right)\leq -\frac{\prod_{\ell =1}^{k}\left(1+d_{\ell }\right)-1}{\prod_{\ell =1}^{k}\left(1-\frac{d_{\ell }}{\lambda_{\min}\left(G_{\ell }\right)}\right)-1}. \end{array}$$

**Proof.**  See Section 4.3.1. □

**Remark** **9.** *As a sanity check, it would be in place to verify that the lower bound on the second-largest eigenvalue in the right-hand side of (52) is smaller than or equal to the largest eigenvalue of the strong product:*

(56) $$\begin{array}{c} \lambda_{\max}\left(G_{1}⊠\ldots ⊠G_{k}\right)=\overset{k}{\prod_{\ell =1}}\left(1+d_{\ell }\right)-1. \end{array}$$

*First, equality (56) holds since $G_{1}⊠\ldots ⊠G_{k}$ is d-regular with a value of d that is equal to the right-hand side of (56). Straightforward algebra reveals that the required inequality we wish to assert readily follows from the inequalities*

(57) $$\begin{array}{c} \theta \left(G_{\ell }\right)\geq \alpha \left(G_{\ell }\right)\geq \frac{n_{\ell }}{1+d_{\ell }},\;\ell \in \left[k\right]. \end{array}$$

*Indeed, the first inequality in (57) holds since the Lovász θ-function of a graph is an upper bound on its graph capacity (see Theorem 1 of [6]), and (by definition) the graph capacity is larger than or equal to the independence number of the graph. The second inequality in (57) holds by Wei’s inequality [80], which provides a lower bound on the independence number or the clique number of a finite simple graph as a function of the degrees of its vertices (see, e.g., page 287 of [64] or page 100 of [81] for a nice probabilistic proof of these inequalities; for some further such bounds, see [82]). For a simple graph G of order n, where vertex i∈[n] is of degree $d_{i}$, Wei’s bound states that*

(58) $$\begin{array}{c} \alpha \left(G\right)\geq \sum_{i=1}^{n}\frac{1}{1+d_{i}},\;\omega \left(G\right)\geq \sum_{i=1}^{n}\frac{1}{n-d_{i}}, \end{array}$$

*so the first inequality in (58) is specialized to the second inequality in (57) for a $d_{\ell }$-regular graph $G_{\ell }$ of order $n_{\ell }$. It should be noted that the pair of inequalities in (57) also imply that, unless not all graphs $\left\{G_{\ell }\right\}$ are empty, the upper bound on $\lambda_{\min}\left(G_{1}⊠\ldots ⊠G_{k}\right)$ in the right-hand side of (54) is smaller than or equal to −1. It is a desired property of this upper bound since the smallest eigenvalue of a non-empty and finite regular graph is smaller than or equal to −1, while attaining this value if the graph is complete.*

**Corollary** **7.** *Let G a d-regular graph of order n. Then, for all k∈N, the second-largest and smallest eigenvalues of the k-fold strong power of G satisfy the inequalities*

(59) $$\begin{array}{c} \lambda_{2}\left(G^{⊠\;k}\right)\geq \frac{n^{k}-{\left(1+d\right)}^{k}}{\theta {\left(G\right)}^{k}-1}-1, \end{array}$$

*and*

(60) $$\begin{array}{c} \lambda_{\min}\left(G^{⊠\;k}\right)\leq -\frac{{\left(1+d\right)}^{k}-1}{{\left(\frac{n}{\theta (G)}\right)}^{k}-1}. \end{array}$$

**Proof.**  See Section 4.3.2. □

**Example** **4.** *By Corollary 7, the second-largest eigenvalue of the k-fold strong power of the 5-cycle (pentagon) graph $C_{5}$ satisfies*

(61) $$\begin{array}{c} \lambda_{2}\left(C_{5}^{⊠\;k}\right)\geq \frac{5^{k}-3^{k}}{5^{\frac{k}{2}}-1}-1,\;k\in N, \end{array}$$

*which holds since $\theta \left(C_{5}\right)=\sqrt{5}$ (it is also the Shannon capacity of the pentagon by Theorem 2 of [6]), and $C_{5}$ is 2-regular (d=2). The lower bound on $\lambda_{2}\left(C_{5}^{⊠\;k}\right)$ in the right-hand side of (61) scales asymptotically (for large k) like $5^{\frac{k}{2}}$. It is next compared with the Alon–Boppana lower bound. The k-fold strong power of $C_{5}$ is a $d_{k}$-regular graph with $d_{k}={\left(1+d\right)}^{k}-1=3^{k}-1$. The Alon–Boppana lower bound in (8) is slightly smaller than $2\sqrt{d_{k}-1}$, which scales asymptotically like $2\cdot 3^{\frac{k}{2}}$. This exemplifies an improvement in the exponential growth rate of the lower bound in (61), as compared to the Alon–Boppana lower bound.*

*We next compare numerically the exact values of $\lambda_{2}\left(C_{5}^{⊠\;k}\right)$, for 1≤k≤5, with their lower bounds in the right-hand side of (61) (the exact values were calculated by the SageMath software [65], and their numerical computation for k>5 seem to be a difficult task). The exact values for k∈[5] are equal to 0.6180,3.8541,13.5623,42.6869,130.0608, respectively, (with 4 digit decimal precision), in comparison to the lower bound in the right-hand side of (61) which is equal to 0.6180,3.0000,8.6264,21.3333 and 51.4938, respectively.*

The next result shows that every non-complete regular graph G, whose Lovász θ-function is below a fixed value, has the property that almost all the strong powers of G are highly non-Ramanujan. The derivation of this result relies on inequality (59).

**Proposition** **3.** *Let G be a non-empty and non-complete connected d-regular graph on n vertices. If*

(62) $$\begin{array}{c} \theta \left(G\right)<\frac{n}{\sqrt{d+1}}, \end{array}$$

*then there exists $k_{0}\in N$ such that, for all $k\geq k_{0}$, the k-fold strong power $G^{⊠\;k}$ is (highly) non-Ramanujan. An explicit closed-form expression for the value of $k_{0}$ is given by*

(63) $$\begin{array}{c} k_{0}=\max\left\{3,\left\lceil \frac{\log\left(2+{\left(d+1\right)}^{-\frac{3}{2}}\right)\;+\log\left(\frac{n^{3}}{n^{3}-{\left(d+1\right)}^{3}}\right)}{\log\left(\frac{n}{\theta \left(G\right)\;\sqrt{d+1}}\right)}\right\rceil \right\}. \end{array}$$

*This holds, in particular, for all finite graphs that are self-complementary and vertex-transitive (they all satisfy the condition in (62) if n>1), with a value of $k_{0}$ which, respectively, is equal to 5, 4 or 3 if n=5, n=9 or n≥13 with n≡1(mod4).*

**Proof.**  See Section 4.3.3. □

**Remark** **10.** *A necessary and sufficient condition for the existence of a self-complementary graph on n vertices is that n≡0(mod4) or n≡1(mod4) (see pages 16–17 of [44], together with a recursive construction of such graphs in Section 2). A self-complementary and d-regular graph of order n satisfies $d=\frac{n-1}{2}$, which implies that n needs to be odd. Since a vertex-transitive graph is regular, the option of n≡0(mod4) is rejected for graphs of order n that are self-complementary and vertex-transitive. This implies that the order n of such graphs must satisfy n≡1(mod4). For n=1 and n=5, there exist graphs of order n that are self-complementary and vertex-transitive; they are, respectively, given by $K_{1}$ and $C_{5}$. Graphs that are self-complementary and vertex-transitive, and approaches for their construction, received attention in the literature (see, e.g., [6,69,83,84,85,86,87]).*

**Remark** **11.** *Proposition 3 was inspired by Example 4, referring to the 5-cycle graph $C_{5}$ that is self-complementary and vertex-transitive. It can be numerically verified, with the SageMath software [65], that $C_{5}$ and $C_{5}^{⊠\;2}$ are Ramanujan graphs, and then the higher strong powers $C_{5}^{⊠\;3}$, $C_{5}^{⊠\;4}$, $C_{5}^{⊠\;5}$ are non-Ramanujan graphs. Proposition 3 shows that all strong powers $C_{5}^{⊠\;k}$, with k≥5, are non-Ramanujan graphs. An expression for $k_{0}$, as it is given in (63), was derived in order to reduce the minimal analytical value of $k_{0}$ for which the k-fold strong power of $C_{5}$ is asserted to be non-Ramanujan for all $k\geq k_{0}$. It started with an initial value of $k_{0}=8$ for n=5, with a more simple initial expression for $k_{0}$, and it was reduced to $k_{0}=5$ with the closed-form expression in (63). Proposition 3, and the numerical experimentation as above, gives that $C_{5}^{⊠\;k}$ is a Ramanujan graph if and only if k=1 or k=2.*

**Example** **5.** *Consider the connected Kneser graphs G=K(m,r) with m>2r and m,r∈N. By their construction, and Theorem 13 of [6],*

(64) n=mr,d=m−rr,θ(G)=m−1r−1.

*The expression of θ(G) in the third equality of (64) relies on the proof of Theorem 13 of [6], which shows that α(G)=Θ(G)=θ(G) (it holds by combining the Erdös-Ko-Rado theorem for finite sets, which serves to determine the independence number of the graph, together with the upper bound on θ(G) in Theorem 9 of [6]). Straightforward algebra shows that the condition in (62) is not necessarily satisfied for the set of parameters in (64). For example, by selecting m=2r+1, the condition in (62) is satisfied if and only if 1≤r≤3 (e.g., it is satisfied by the Petersen graph, which corresponds to r=2). The condition in (62) is violated for r≥4 so, for these values of the parameter r, the exponential growth rate of the Alon–Boppana lower bound on $\lambda_{2}\left(G^{⊠\;k}\right)$ is larger than the exponent of the suggested lower bound in the right-hand side of (59).*

**Remark** **12.** *Several methods for obtaining upper bounds on the smallest eigenvalue of a graph G rely on the identity $\lambda_{\min}\left(G\right)=\min_{x\neq 0}\frac{x^{T}Ax}{x^{T}x}$ (see, e.g., [88]). The dimensions of the adjacency matrix of a k-fold strong power of a graph G grow exponentially in k, so the computational complexity of such bounds is typically very high for strong graph powers. However, for strong graph powers, the eigenvalue bounds in Corollary 7 are analytical, and their computational complexity is not affected by k. The reader is also referred to recent works which derive lower bounds on the smallest eigenvalue of a graph based on graph decompositions [89], and lower bounds on the smallest eigenvalue of regular graphs containing many copies of a smaller fixed subgraph [90]. Such lower bounds on the smallest eigenvalue of a regular graph transform to upper bounds on the second-largest eigenvalue of regular graphs by using equality (5). Some upper bounds on the second-largest eigenvalue of connected graphs, with conditions for their attainability, are provided in [91]. The paper [92] surveys (less recent) bounds on the eigenvalues of simple graphs.*

### 3.4. Lower Bounds on the Chromatic Numbers of Strong Products

The problem of relating the chromatic number of a graph to its eigenvalues dates back to Haemers (Section 2.2 of [74]), Hoffman [93], and Wilf [94], obtaining upper and lower bounds on the chromatic numbers of graphs in terms of their largest, second-largest and smallest eigenvalues.

This section presents lower bounds on the chromatic number of a strong product of graphs (and its complement) in terms of the Lovász θ-functions (or smallest eigenvalues) of its factors.

**Proposition** **4.** *The following lower bounds on chromatic numbers of strong products hold:*

*(a)*  *Let $G_{1},\ldots ,G_{k}$ be k simple graphs, $\left|V(G_{\ell })\right|=n_{\ell }$ for ℓ∈[k], and $G=G_{1}⊠\ldots ⊠G_{k}$. Then,*
(65) $$\begin{array}{cc} \; & \chi \left(G\right)\geq \left\lceil \prod_{\ell =1}^{k}\frac{n_{\ell }}{\theta (G_{\ell })}\right\rceil , \end{array}$$
(66) $$\begin{array}{cc} \; & \chi \left(\bar{G}\right)\geq \left\lceil \prod_{\ell =1}^{k}\theta \left(G_{\ell }\right)\right\rceil . \end{array}$$
*(b)*  *Let $G_{1},\ldots ,G_{k}$ be regular graphs, where $G_{\ell }$ is $d_{\ell }$-regular of order $n_{\ell }$ for all ℓ∈[k]. Then,*
(67) $$\begin{array}{cc} \chi (G) & \geq \left\lceil \prod_{\ell =1}^{k}\frac{n_{\ell }}{\theta (G_{\ell })}\right\rceil \end{array}$$
(68) $$\begin{array}{cc} \; & \geq \left\lceil \prod_{\ell =1}^{k}\left(1-\frac{d_{\ell }}{\lambda_{\min}\left(G_{\ell }\right)}\right)\right\rceil , \end{array}$$
*and inequality (68) holds with equality if each $G_{\ell }$ is either edge-transitive or strongly regular.*
*(c)*  *If, for all ℓ∈[k], $G_{\ell }$ is $d_{\ell }$-regular, and it is either edge-transitive or strongly regular, then*
(69) $$\begin{array}{c} \prod_{\ell =1}^{k}\left(1-\frac{d_{\ell }}{\lambda_{\min}\left(G_{\ell }\right)}\right)\geq 1-\frac{d(G)}{\lambda_{\min}\left(G\right)}, \end{array}$$
*where*
(70) $$\begin{array}{c} d\left(G\right)=\prod_{\ell =1}^{k}\left(1+d_{\ell }\right)-1 \end{array}$$
*is the valency of the regular graph $G=G_{1}⊠\ldots ⊠G_{k}$, and $\lambda_{\min}\left(G\right)$ is its smallest eigenvalue.*
*(d)*  *Let $G_{1},\ldots ,G_{k}$ be regular graphs, where $G_{\ell }$ is $d_{\ell }$-regular of order $n_{\ell }$ for all ℓ∈[k].*
  *(1)*  *If, for all ℓ∈[k], the graph $G_{\ell }$ is either vertex-transitive or strongly regular, then the lower bound on χ(G) in the right-hand side of (65) is larger than or equal to the lower bound $\prod_{\ell =1}^{k}\omega \left(G_{\ell }\right)$.*
  *(2)*  *If, for all ℓ∈[k], the graph $G_{\ell }$ is either (i) both vertex-transitive and edge-transitive, or (ii) strongly regular, then the lower bound on χ(G) in the right-hand side of (68) is larger than or equal to the lower bound $\prod_{\ell =1}^{k}\omega \left(G_{\ell }\right)$.*
*(e)*  *Let, for all ℓ∈[k], the graph $G_{\ell }$ be $d_{\ell }$-regular on $n_{\ell }$ vertices, and suppose that it is either edge-transitive or strongly regular. Then,*
(71) $$\begin{array}{c} \chi \left(\bar{G}\right)\geq \left\lceil \prod_{\ell =1}^{k}\left(-\frac{n_{\ell }\;\lambda_{\min}\left(G_{\ell }\right)}{d_{\ell }-\lambda_{\min}\left(G_{\ell }\right)}\right)\right\rceil . \end{array}$$
*(f)*  *Let, for all ℓ∈[k], $G_{\ell }$ be a self-complementary graph on $n_{\ell }$ vertices that is either vertex-transitive or strongly regular. Let $n≜\prod_{\ell =1}^{k}n_{\ell }$ be the order of $G=G_{1}⊠\ldots ⊠G_{n}$. Then,*
(72) $$\begin{array}{ccc} & & \chi \left(G\right)\geq \left\lceil \sqrt{n}\;\right\rceil , \end{array}$$
(73) $$\begin{array}{ccc} & & \chi \left(\bar{G}\right)\geq \left\lceil \sqrt{n}\;\right\rceil . \end{array}$$

**Proof.**  See Section 4.4.1. □

**Remark** **13.** *The following inequality is proved in Theorem 11 of [14]:*

(74) $$\begin{array}{c} \chi \left({\bar{G}}_{1}⊠{\bar{G}}_{2}\right)\geq \theta \left(G_{1}\right)\;\theta \left(G_{2}\right). \end{array}$$

*(Analogous inequalities to (74) were derived by Hales [27], and by McEliece and Posner [34]; see Theorem 10 of [14]). Inequality (65) can be obtained by combining (17), (18) and (74). This can be done by first replacing $G_{1}$ and $G_{2}$ in (74) with $G_{1}^{\prime }≜\bar{G_{1}}$ and $G_{2}^{\prime }≜\bar{G_{2}⊠\ldots ⊠G_{k}}$, respectively, then relying on (18) to get the inequalities $\theta \left(G_{1}^{\prime }\right)\geq \frac{n_{1}}{\theta (G_{1})}$ and $\theta \left(G_{2}^{\prime }\right)\geq \frac{n_{2}\ldots n_{k}}{\theta (\bar{G_{2}^{\prime }})}$, and finally relying on (17) to get the equality $\theta \left(\bar{G_{2}^{\prime }}\right)=\theta \left(G_{2}\right)\ldots \theta \left(G_{k}\right)$. Our two simple proofs of (65) are, however, easier than this one, and they do not require to rely on (74).*

**Remark** **14.** *Inequality (74) differs from (66), although the lower bounds in the right-hand sides are similar for k=2. The difference between (66) and (74) is that the left-hand side of (66) refers to the chromatic number of $\bar{G_{1}⊠G_{2}}$, whereas the left-hand side of (74) refers to $\bar{G_{1}}⊠\bar{G_{2}}$. The result in (66) appears in Proposition 4 for completeness since it is an immediate consequence of the sandwich theorem in (15), and the identity in (17).*

**Remark** **15.** *Under the assumptions of Item (c) in Proposition 4, the lower bound on χ(G) in the right-hand side of (68) is larger than or equal to Hoffman’s lower bound (see (69)). This holds in addition to the high complexity in computing $\lambda_{\min}\left(G\right)$ in the right-hand side of (69), even for relatively small values of k. Hoffman’s bound was originally proved for regular graphs [93], and it was later extended by Haemers to general simple and finite graphs (see Proposition 3.5.3 of [39], Theorem 2.1.3 of [74], Corollary 8.10 and Theorem 8.11 of [43]), and to hypergraphs [95]. A recent perspective on Hoffman’s bound appears in [96].*

**Remark** **16.** *We refer to Item (f) of Proposition 4. If G is self-complementary of order n, then*

(75) $$\begin{array}{c} \chi \left(G\right)\geq \left\lceil \sqrt{n}\;\right\rceil . \end{array}$$

*Indeed, for every graph G,*

(76) $$\begin{array}{cc} \chi \left(G\right)\;\chi \left(\bar{G}\right) & \geq \theta \left(G\right)\;\theta \left(\bar{G}\right) \end{array}$$

(77) $$\begin{array}{cc} \; & \geq n, \end{array}$$

*where inequality (76) holds since, by (15) and (16), $\chi \left(G\right)\geq \theta \left(\bar{G}\right)$ and $\chi \left(\bar{G}\right)\geq \theta \left(G\right)$, and inequality (77) holds by Corollary 2 of [6]. The chromatic number is invariant under isomorphism, so $\chi \left(G\right)=\chi \left(\bar{G}\right)$ if G is self-complementary. This gives (75) from (77). It should be noted, however, that the result in Item (f) (see (72) and (73)) is not implied by (75). This is because a strong product of self-complementary graphs, where each factor is also either vertex-transitive or strongly regular, does not necessarily give a self-complementary graph. As a counter example, let $G_{1}=G_{2}=C_{5}$. The pentagon $C_{5}$ is a self-complementary, vertex-transitive and strongly regular graph, whereas $G=C_{5}⊠C_{5}$ is not a self-complementary graph by Theorem 1.1 of [84]; according to that theorem, G would have been self-complementary if the strong product of $G_{1}$ and $G_{2}$ had been replaced by their lexicographic product (the last two observations were also verified by the SageMath software [65]).*

The next result specializes Item (a) of Proposition 4 (see (65)) to strong products of strongly regular graphs. This result is obtained by relying on Corollary 1 that provides a closed-form expression for the Lovász θ-function of each factor.

**Corollary** **8.** *Let $G_{1},\ldots ,G_{k}$ be strongly regular graphs with parameters $\mathrm{srg}(n_{\ell },d_{\ell },\lambda_{\ell },\mu_{\ell })$ for ℓ∈[k] (they need not be distinct). Then, the chromatic number of their strong product satisfies*

(78) $$\begin{array}{ccc} & & \left\lceil \prod_{\ell =1}^{k}\left(1+\frac{2d_{\ell }}{t_{\ell }+\mu_{\ell }-\lambda_{\ell }}\right)\right\rceil \leq \chi \left(G_{1}⊠\ldots ⊠G_{k}\right)\leq \prod_{\ell =1}^{k}\chi \left(G_{k}\right), \end{array}$$

*where ${\left\{t_{\ell }\right\}}_{\ell =1}^{k}$ in the leftmost term of (78) is given by*

(79) $$\begin{array}{c} t_{\ell }≜\sqrt{{\left(\lambda_{\ell }-\mu_{\ell }\right)}^{2}+4\left(d_{\ell }-\mu_{\ell }\right)},\;\ell \in \left[k\right]. \end{array}$$

*The leftmost term in (78) is also larger than or equal to the product of the clique numbers of the factors ${\left\{G_{\ell }\right\}}_{\ell =1}^{k}$.*

**Proof.**  See Section 4.4.2. □

The next two examples present strong products of strongly regular graphs, whose chromatic numbers are exactly determined by Corollary 8.

**Example** **6.** *Let $G_{1},G_{2},G_{3}$ be the Schläfli, Shrikhande, and Hall-Janko graphs, respectively. These are strongly regular graphs whose parameters are srg(27,16,10,8), srg(16,6,2,2), and srg(100,36,14,12), respectively. Their chromatic numbers are equal to $\chi (G_{1})=9$, $\chi (G_{2})=4$, and $\chi (G_{3})=10$. Consider the chromatic number of the strong product of arbitrary nonnegative powers of $G_{1}$, $G_{2}$ and $G_{3}$. It can be verified that, for all such strong products, the upper and lower bounds in Corollary 8 coincide, so for all integers $k_{1},k_{2},k_{3}\geq 0$,*

(80) $$\begin{array}{c} \chi \left(G_{1}^{⊠\;k_{1}}⊠G_{2}^{⊠\;k_{2}}⊠G_{3}^{⊠\;k_{3}}\right)=9^{k_{1}}\;4^{k_{2}}\;10^{k_{3}}. \end{array}$$

*For comparison, the lower bound that is given by the product of the clique numbers of each factor is equal to $6^{k_{1}}3^{k_{2}}4^{k_{3}}$ (since $\omega (G_{1})=6$, $\omega (G_{2})=3$, and $\omega (G_{3})=4$). This shows that it is significantly looser than the tight lower bound in the right-hand side of (80).*

**Example** **7.** *Consider the three non-isomorphic Chang graphs. These are strongly regular graphs with the same set of parameters srg(28,12,6,4). The clique number of one of these graphs is equal to 5, and the clique numbers of the other two graphs are equal to 6. Let us denote these graphs by $G_{1}$, $G_{2}$ and $G_{3}$, such that $\omega (G_{1})=5$, $\omega (G_{2})=6$, and $\omega (G_{3})=6$. The chromatic numbers of all these three graphs are similar, and they are equal to 7, i.e., $\chi \left(G_{1}\right)=\chi \left(G_{2}\right)=\chi \left(G_{3}\right)=7$ (the clique and chromatic numbers of the three Chang graphs are easy to verify with the SageMath software [65]). Let $k_{1},k_{2}$ and $k_{3}$ be arbitrary nonnegative integers. By Corollary 8,*

(81) $$\begin{array}{c} \chi \left(G_{1}^{⊠\;k_{1}}⊠G_{2}^{⊠\;k_{2}}⊠G_{3}^{⊠\;k_{3}}\right)=7^{k_{1}+k_{2}+k_{3}} \end{array}$$

*due to the coincidence of the upper and lower bounds in (78). For comparison, the lower bound on the chromatic number in the left-hand side of (81), which is given by the product of the clique numbers of each factor, is equal to $5^{k_{1}}6^{k_{2}+k_{3}}$.*

The next example presents a power product of a vertex and edge-transitive regular graph, whose chromatic number is exactly determined by Item (b) of Proposition 4.

**Example** **8.** *The present example provides the exact value of $\chi (G^{⊠\;k})$ where G is the Perkel graph, and k∈N. The Perkel graph is 6-regular on 57 vertices, and it is both vertex-transitive and edge-transitive. The clique and chromatic numbers of G are equal to ω(G)=2 and χ(G)=3, respectively, and the smallest eigenvalue of (the adjacency matrix of) G is equal to $\lambda_{\min}\left(G\right)=-3$. By Item (b) of Proposition 4, for all k∈N,*

(82) $$\begin{array}{c} \chi \left(G^{⊠\;k}\right)\geq {\left(1-\frac{d}{\lambda_{\min}\left(G\right)}\right)}^{k}=3^{k}, \end{array}$$

*which can be compared here to the simple upper and lower bounds on $\chi (G^{⊠\;k})$ whose values are given by $\chi {\left(G\right)}^{k}=3^{k}$ and $\omega {\left(G\right)}^{k}=2^{k}$, respectively. The coincidence of the improved lower bound in (82) and the upper bound gives that, for all k∈N,*

(83) $$\begin{array}{c} \chi \left(G^{⊠\;k}\right)=3^{k}. \end{array}$$

The next two examples illustrate numerically Part 2 of Item (d) in Proposition 4.

**Example** **9.** *Let G be the Suzuki graph, which is a strongly regular graph with parameters srg(1782,416,100,96) (see Section 10.83 of [60]). The lower bound in Corollary 8 (see the leftmost term in (78)) gives that $\chi \left(G^{⊠\;k}\right)\geq 27^{k}$ for all k∈N. For comparison, since ω(G)=6, the lower bound that is based on the clique number of G gives $\chi \left(G^{⊠\;k}\right)\geq 6^{k}$ for all k∈N. The exact value of χ(G) is not available for the Suzuki graph, so the upper bound in the rightmost term of (78) is unknown. The improvement in the exponential lower bound on the chromatic number of the strong powers $G^{⊠\;k}$ is, however, significant since it is increased from $6^{k}$ to $27^{k}$.*

**Example** **10.** *Let G be the Gosset graph. We apply here Item (b) of Proposition 4 in order to obtain an improved lower bound on $\chi (G^{⊠\;k})$ for all k∈N. The graph G is 27-regular on 56 vertices (i.e., d=27 and n=56); it is both vertex-transitive and edge-transitive, and it is also not strongly regular. The clique and chromatic numbers of G are equal to ω(G)=7 and χ(G)=14, respectively, and the smallest eigenvalue of (the adjacency matrix of) G is equal to $\lambda_{\min}\left(G\right)=-3$. By Item (b) of Proposition 4 (note that the edge-transitivity of G implies that (68) holds with equality), it follows that for all k∈N,*

(84) $$\begin{array}{cc} \chi (G^{⊠\;k}) & \geq {\left(1-\frac{d}{\lambda_{\min}\left(G\right)}\right)}^{k} \end{array}$$

(85) $$\begin{array}{cc} \; & =10^{k}, \end{array}$$

*which can be compared here to the simple upper and lower bounds on $\chi (G^{⊠\;k})$ whose values are given by $\chi {\left(G\right)}^{k}=14^{k}$ and $\omega {\left(G\right)}^{k}=7^{k}$, respectively.*

All the factors of the strong products in Examples 6–10 are either strongly regular or otherwise, they are both vertex-transitive and edge-transitive graphs. Consequently, by Item (b) of Proposition 4, the two lower bounds in the right-hand sides of (67) and (68) coincide. Furthermore, by Part 2 of Item (d) in Proposition 4, they also offer an improvement over the simple lower bound that is equal to the product of the clique numbers of each factor of the strong product. The next example shows that such an improvement does not necessarily take place if the factors of the strong product of the regular graphs are not vertex-transitive and edge-transitive, while also not being strongly regular.

**Example** **11.** *Let G be the Frucht graph, which is 3-regular on 12 vertices (i.e., d=3 and n=12). This graph is not strongly regular, and also not vertex-transitive or edge-transitive. The clique and chromatic numbers of this graph are both equal to 3 (i.e., ω(G)=χ(G)=3), so*

(86) $$\begin{array}{c} \chi \left(G^{⊠\;k}\right)=3^{k},\;k\in N. \end{array}$$

*The smallest eigenvalue of the adjacency matrix of G is equal to $\lambda_{\min}\left(G\right)=-2.33866$. By (68), for all k∈N,*

(87) $$\begin{array}{cc} \chi (G^{⊠\;k}) & \geq {\left(1-\frac{d}{\lambda_{\min}\left(G\right)}\right)}^{k} \end{array}$$

(88) $$\begin{array}{cc} \; & =2.28278^{k}. \end{array}$$

*This shows that the lower bound on the chromatic number in the right-hand side of (68) may be looser than the simple lower bound that is equal to the product of the clique numbers of each factor. This may happen if some of the factors, which are regular graphs, are not either strongly regular, or both vertex-transitive and edge-transitive.*

### 3.5. The Shannon Capacity of Strongly Regular Graphs

The Lovász θ-function of a strongly regular graph is expressed in closed-form in Corollary 1, being also an upper bound on the Shannon capacity of such a graph (by Theorem 1 of [6]). The independence number of a graph is, on the other hand, a lower bound on the Shannon capacity. The Shannon capacity of such a graph is therefore determined if these upper and lower bounds coincide. The following examples show such cases of coincidence for strongly regular graphs. The first one reproduces (in a different way) the Shannon capacity of the Petersen graph, a result dating back to Lovász [6]. The rest of the examples provide new results that determine the Shannon capacity of some strongly regular graphs.

**Example** **12.** *Let G be the Petersen graph, whose Shannon capacity is equal to 4. It is a special case of Theorem 13 of [6], which determines the Shannon capacity of Kneser graphs*

(89) ΘK(m,r)=m−1r−1,m≥2r.

*Then, the capacity of the Petersen graph is obtained in Corollary 6 of [6] by viewing it as an isomorphic graph to the Kneser graph K(5,2).*

*As an alternative way to determine its Shannon capacity, the Petersen graph is a strongly regular graph with parameters srg(10,3,0,1) (see Section 10.3 of [60]). By Corollary 1, it follows that θ(G)=4. This coincides with the independence number α(G)=4, so Θ(G)=4.*

**Example** **13.** *Let G be the Shrikhande graph, which is strongly regular with the parameters srg(16,6,2,2) (see Section 10.6 of [60]). By Corollary 1, it can be verified that θ(G)=4. Its chromatic number is χ(G)=4, and its independence number is α(G)=4 (so α(G)χ(G)=n, which implies that the n=16 vertices in G are partitioned into four color classes, where each color class is a largest independent set in G of size 4). Hence, the graph capacity is equal to Θ(G)=4.*

*The capacity of every graph G of order n that is self-complementary and vertex-transitive is $\Theta \left(G\right)=\sqrt{n}$ (see Theorem 12 of [6]). It should be noted, however, that although the capacity of the Shrikhande graph is a case where $\Theta \left(G\right)=\sqrt{n}$ (with n=16), it does not follow from Theorem 12 of [6] since the Shrikhande graph is vertex-transitive, but it is not self-complementary.*

**Example** **14.** *Consider the Hall-Janko graph G, which is a strongly regular graph whose parameters are given by srg(100,36,14,12) (see Section 10.32 of [60]). By Corollary 1, θ(G)=10. The independence number of this graph is α(G)=10 (this was obtained by the SageMath software [65]). Hence, the Shannon capacity of the Hall-Janko graph is Θ(G)=10. The graph G is vertex-transitive but it is not self-complementary so, similarly to Example 13, this result does not follow from the capacity result in Theorem 12 of [6] for finite graphs that are vertex-transitive and self-complementary.*

**Example** **15.** *The Hoffman-Singleton graph G is a strongly regular graph with parameters srg(50,7,0,1) (see Section 10.19 of [60]). By Corollary 1, θ(G)=15. The independence number of this graph is α(G)=15, so the Shannon capacity of this graph is Θ(G)=15.*

**Example** **16.** *The Schläfli graph G is a strongly regular graph with parameters srg(27,16,10,8) (see Section 10.10 of [60]). By Corollary 1, it follows that θ(G)=3. The independence number of this graph is α(G)=3, so the Shannon capacity of this graph is Θ(G)=3.*

**Example** **17.** *The Sims-Gewirtz graph G is a strongly regular graph with parameters srg(56,10,0,2) (also known as the Gewirtz graph) (see Section 10.20 of [60]). By Corollary 1, θ(G)=16, and also α(G)=16 (it can be verified by the SageMath software [65]). The Shannon capacity of the Sims-Gewirtz graph is therefore equal to Θ(G)=16.*

**Example** **18.** *The $M_{22}$ graph (also known as the Mesner graph) G is a strongly regular graph with parameters srg(77,16,0,4) (see Section 10.27 of [60]). Its independence number is α(G)=21, and by Corollary 1 also θ(G)=21. Consequently, the Shannon capacity of the $M_{22}$ graph is Θ(G)=21.*

**Example** **19.** *The Cameron graph G is strongly regular with parameters srg(231,30,9,3) (see Section 10.54 of [60]). By Corollary 1, θ(G)=21, which coincides with the independence number of G, i.e., α(G)=21. The Shannon capacity of the Cameron graph is, hence, equal to Θ(G)=21.*

**Example** **20.** *Consider the three non-isomorphic Chang graphs. These are strongly regular graphs with the same set of parameters srg(28,12,6,4) (see Section 10.11 of [60]). By Corollary 1, the Lovász θ-function of these graphs is equal to 4, which coincides with their independence number. The Shannon capacity of each of these three graphs is therefore equal to 4.*

**Remark** **17.** *Examples 12–20 provide several strongly regular graphs for which their Lovász θ-function coincides with their independence number, thus determining the Shannon capacity of these graphs. This, however, is not the case in general for strongly regular graphs. For example, in continuation to Example 16, the Lovász θ-function of the complement of the Schläfli graph $\bar{G}$ (it is a strongly regular graph srg(27,10,1,5)) is equal to $\theta (\bar{G})=9$. Indeed, $\theta \left(G\right)\;\theta \left(\bar{G}\right)=27$ is the order of G. It was proved, however, by the rank-bound of Haemers [7] that the capacity of the complement of the Schläfli graph is at most 7. It can be also verified that the independence number of $\bar{G}$ (i.e., the clique number of the Schläfli graph G) is equal to 6. This overall gives that $6=\omega \left(G\right)\leq \Theta \left(\bar{G}\right)\leq 7<9=\theta \left(\bar{G}\right)$.*

We end this section by studying some capacity results of affine polar graphs.

**Example** **21.** *Affine polar graphs (see, e.g., Section 3.3 of [60]) are constructed by considering a vector space V of dimension d over a finite field $F_{q}$ with q elements, equipped with a non-degenerate quadratic form Q. The vertices in the graph are represented by the vectors in V, and any two vectors u and v (i.e., a pair of vertices in the graph) are adjacent if Q(u−v)=0. The resulting graph is denoted by ${\mathrm{VO}}^{+}\left(d,q\right)$, ${\mathrm{VO}}^{-}\left(d,q\right)$, and VO(d,q) when the quadratic form Q is hyperbolic, elliptic or parabolic, respectively. In the first two cases, d is even, and in the third case, d is odd; in all cases, q is a positive integral power of a prime number (as it is the cardinality of the finite field $F_{q}$). Under these conditions on d and q, the graphs ${\mathrm{VO}}^{+}\left(d,q\right)$ and ${\mathrm{VO}}^{-}\left(d,q\right)$ are strongly regular. Let d=2e with e∈N. The parameters of $G^{+}={\mathrm{VO}}^{+}\left(2e,q\right)$, which is a strongly regular graph $\mathrm{srg}(n^{+},d^{+},\lambda^{+},\mu^{+})$, are given by (see Section 3.3 of [60])*

(90) $$\begin{array}{ccc} & & n^{+}=q^{2e}, \end{array}$$

(91) $$\begin{array}{ccc} & & d^{+}=\left(q^{e-1}+1\right)\left(q^{e}-1\right), \end{array}$$

(92) $$\begin{array}{ccc} & & \lambda^{+}=q\left(q^{e-2}+1\right)\left(q^{e-1}-1\right)+q-2, \end{array}$$

(93) $$\begin{array}{ccc} & & \mu^{+}=q^{e-1}\left(q^{e-1}+1\right), \end{array}$$

(94) $$\begin{array}{ccc} & & \lambda_{2}\left(G^{+}\right)=q^{e}-q^{e-1}-1, \end{array}$$

(95) $$\begin{array}{ccc} & & \lambda_{n^{+}}\left(G^{+}\right)=-q^{e-1}-1, \end{array}$$

*and $G^{-}={\mathrm{VO}}^{-}\left(2e,q\right)$, a strongly regular graph $\mathrm{srg}(n^{-},d^{-},\lambda^{-},\mu^{-})$, has the parameters*

(96) $$\begin{array}{ccc} & & n^{-}=q^{2e}, \end{array}$$

(97) $$\begin{array}{ccc} & & d^{-}=\left(q^{e-1}-1\right)\left(q^{e}+1\right), \end{array}$$

(98) $$\begin{array}{ccc} & & \lambda^{-}=q\left(q^{e-2}-1\right)\left(q^{e-1}+1\right)+q-2, \end{array}$$

(99) $$\begin{array}{ccc} & & \mu^{-}=q^{e-1}\left(q^{e-1}-1\right), \end{array}$$

(100) $$\begin{array}{ccc} & & \lambda_{2}\left(G^{-}\right)=q^{e-1}-1, \end{array}$$

(101) $$\begin{array}{ccc} & & \lambda_{n^{-}}\left(G^{-}\right)=-q^{e}+q^{e-1}-1. \end{array}$$

*By Corollary 1, combined with the parameters (and eigenvalues) in (90)–(101), it follows that*

(102) $$\begin{array}{ccc} & & \theta \left(G^{+}\right)=q^{e}=\sqrt{n^{+}}, \end{array}$$

(103) $$\begin{array}{ccc} & & \theta \left(\bar{G^{+}}\right)=q^{e}, \end{array}$$

(104) $$\begin{array}{ccc} & & \theta \left(G^{-}\right)=q\left(q^{e}-q^{e-1}+1\right), \end{array}$$

(105) $$\begin{array}{ccc} & & \theta \left(\bar{G^{-}}\right)=\frac{q^{2e-1}}{q^{e}-q^{e-1}+1}. \end{array}$$

*Consequently, it can be verified that for some of these affine polar graphs (with the free parameters q and e as above), their Lovász θ-function and independence number coincide. (The numerical computations of the independence numbers of these graphs were performed by the SageMath software [65].) This gives, for example, that*

(106) $$\begin{array}{ccc} & & \Theta \left({\mathrm{VO}}^{+}\left(4,2\right)\right)=4,\;\Theta \left({\mathrm{VO}}^{+}\left(6,2\right)\right)=8, \end{array}$$

(107) $$\begin{array}{ccc} & & \Theta \left({\mathrm{VO}}^{+}\left(4,3\right)\right)=9,\;\Theta \left({\mathrm{VO}}^{+}\left(6,3\right)\right)=27, \end{array}$$

*as the exact values of the Shannon capacities of these strongly regular graphs, whose parameters are, respectively, srg(16,9,4,6), srg(64,35,18,20), srg(81,32,13,12), and srg(729,260,97,90).*

## 4. Proofs

### 4.1. Proofs for Section 3.1

#### 4.1.1. Proof of Proposition 1

Let G be a *d*-regular graph with *n* vertices. The rightmost inequality in (24) is provided in Theorem 9 of [6], together with a sufficient condition that it holds with equality if G is edge-transitive. Another sufficient condition for that inequality to hold with equality is obtained later in this proof.

We first prove the leftmost inequality in (24), and also obtain sufficient conditions that it holds with equality. By (18) (see Corollary 2 of [6]),

(108) $$\begin{array}{c} \theta \left(G\right)\geq \frac{n}{\theta (\bar{G})} \end{array}$$

and, by Theorem 8 of [6], equality holds in (108) if G is vertex-transitive (or, equivalently, if $\bar{G}$ is vertex-transitive). Since $\bar{G}$ is an (n−d−1)-regular graph of order *n*, an application of (19) (see Theorem 9 of [6]) to $\bar{G}$ gives

(109) $$\begin{array}{c} \theta \left(\bar{G}\right)\leq -\frac{n\lambda_{n}\left(\bar{G}\right)}{\left(n-d-1\right)-\lambda_{n}\left(\bar{G}\right)}, \end{array}$$

with equality in (109) if $\bar{G}$ is edge-transitive. Combining (108) and (109) gives

(110) $$\begin{array}{c} \theta \left(G\right)\geq 1-\frac{n-d-1}{\lambda_{n}\left(\bar{G}\right)}, \end{array}$$

with equality in (110) if $\bar{G}$ is vertex-transitive and edge-transitive (recall that a vertex-transitive graph is regular). By the regularity of G, we have from (5),

(111) $$\begin{array}{c} \lambda_{n}\left(\bar{G}\right)=-1-\lambda_{2}\left(G\right). \end{array}$$

Combining (110) and (111) gives the leftmost inequality in (24), together with the conclusion that it holds with equality if $\bar{G}$ is both vertex-transitive and edge-transitive. Next, (25) is obtained from (24) by replacing G with $\bar{G}$, and relying on the equality in (111). A sufficient condition that the leftmost inequality in (25) holds with equality is therefore the same condition that the leftmost inequality in (24) holds with equality, while replacing G with $\bar{G}$; this means that the leftmost inequality in (25) holds with equality if G is vertex-transitive and edge-transitive. Likewise, the rightmost inequality in (25) holds with equality under the same condition that the rightmost inequality in (24) holds with equality, while the graph G in (24) is replaced by its complement $\bar{G}$ in (25). This means that the rightmost inequality in (24) holds with equality if $\bar{G}$ is edge-transitive (recall that, unlike vertex-transitivity that is a property of G if and only if it is a property of its complement $\bar{G}$, it is not the case for edge-transitivity).

We finally prove that if G is a strongly regular graph, then the four inequalities in (24) and (25) hold with equalities. Let G be a strongly regular graph srg(n,d,λ,μ). Then, the largest, second-largest, and smallest eigenvalues of G are given by

(112) $$\begin{array}{ccc} & & \lambda_{1}\left(G\right)=d, \end{array}$$

(113) $$\begin{array}{ccc} & & \lambda_{2}\left(G\right)=r≜\frac{1}{2}\;\left[\lambda -\mu +\sqrt{{\left(\lambda -\mu \right)}^{2}+4\left(d-\mu \right)}\right], \end{array}$$

(114) $$\begin{array}{ccc} & & \lambda_{n}\left(G\right)=s≜\frac{1}{2}\;\left[\lambda -\mu -\sqrt{{\left(\lambda -\mu \right)}^{2}+4\left(d-\mu \right)}\right], \end{array}$$

where $p_{1,2}$ in (10) are replaced here with *r* and *s* in (113) and (114), respectively. (Recall that if G is a disconnected strongly regular graph, then μ=0 and λ=d−1 by (9), which then indeed gives that $\lambda_{2}\left(G\right)=d=\lambda_{1}\left(G\right)$ since the *d*-regular graph is disconnected). The substitution of (113) and (114) into (24) and (25) gives

(115) $$\begin{array}{c} \frac{n-d+r}{1+r}\leq \theta \left(G\right)\leq -\frac{ns}{d-s}, \end{array}$$

and

(116) $$\begin{array}{c} 1-\frac{d}{s}\leq \theta \left(\bar{G}\right)\leq \frac{n(1+r)}{n-d+r}. \end{array}$$

We show as follows that the rightmost and leftmost terms in (115) coincide, turning the two inequalities in (115) into equalities. The coincidence of the rightmost and leftmost terms in (115) is equivalent to proving the equality

(117) $$\begin{array}{c} (n-d+r)\;(d-s)+ns\;(1+r)=0. \end{array}$$

Indeed, we get

$$\begin{array}{ccc} & & \left(n-d+r)\;(d-s)+ns\;(1+r\right) \end{array}$$

(118) $$\begin{array}{ccc} & & \;=nd-d^{2}+d\;\left(r+s\right)+\left(n-1\right)rs \end{array}$$

(119) $$\begin{array}{ccc} & & \;=nd-d^{2}+d\;\left(\lambda -\mu \right)+\left(n-1\right)\;\left(\mu -d\right) \end{array}$$

(120) $$\begin{array}{ccc} & & \;=-d\;(d-\lambda -1)+\mu \;(n-d-1) \end{array}$$

(121) $$\begin{array}{ccc} & & \;=0, \end{array}$$

where (118) and (120) hold by straightforward algebra; (119) holds by (113) and (114); (121) holds by the identity in (9).

The same conclusion also holds for the rightmost and leftmost terms in (116), which can be shown to coincide if G is a strongly regular graph. This turns the two inequalities in (116) into equalities. It holds in a similar way to the previous part since the complement of a strongly regular graph is also strongly regular, and the second-largest and smallest eigenvalues of G in (115) (i.e., r,s respectively) are replaced in (115) by the second-largest and smallest eigenvalues of $\bar{G}$ (i.e., −1−s and −1−r respectively). This proves that each of the four inequalities in (24) and (25) holds with equality if G is a strongly regular graph.

#### 4.1.2. Proof of Corollary 1

Let G be a strongly regular graph srg(n,d,λ,μ). By Proposition 1,

(122) $$\begin{array}{cc} \theta (G) & =\frac{n-d+r}{1+r} \end{array}$$

(123) $$\begin{array}{cc} \; & =-\frac{ns}{d-s}, \end{array}$$

and

(124) $$\begin{array}{cc} \theta (\bar{G}) & =1-\frac{d}{s} \end{array}$$

(125) $$\begin{array}{cc} \; & =\frac{n(1+r)}{n-d+r}, \end{array}$$

where *r* and *s* are given in (113) and (114), respectively. Their substitution into the right-hand sides of (123) and (124) gives (26) and (28), respectively, with the auxiliary parameter *t* as defined in (29). Equality (27) is trivial by (122)–(125). This verifies (26)–(28) for all strongly regular graphs.

Since the multiplicities of the distinct eigenvalues of a strongly regular graph G are integers, it follows from (11) that if 2d+(n−1)(λ−μ)≠0, then *t* in (29) should be an integer. This implies that, under the latter condition, θ(G) and $\theta (\bar{G})$ are necessarily rational numbers.

### 4.2. Proofs for Section 3.2

#### 4.2.1. Proof of Corollary 2

Let G be a non-complete, *d*-regular graph of order *n*. Straightforward algebra gives (30) by comparing the rightmost and leftmost terms in (24). Inequalities (30) and (31) can be verified to be equivalent by relying on the inequality $d+\left(n-1\right)\lambda_{n}\left(G\right)<0$ (this holds since $\lambda_{n}\left(G\right)\leq -1$, and d<n−1 for a non-complete, *d*-regular graph G with *n* vertices). Equality holds in (30) if and only if the rightmost and leftmost terms in (24) are equal to θ(G) (i.e., they are both equal to the middle term in (24)). According to Item (a) of Proposition 1, equality holds in (30) and (31) if G is strongly regular.

We next show that the latter sufficient condition is also necessary. To that end, we provide a second proof that relies on linear algebra. Let A=A(G) and $\bar{A}=A\left(\bar{G}\right)$ be the adjacency matrices of G and $\bar{G}$, respectively. Let $J^{\left(n\right)}$ and $I_{n}$ denote the *n*-times-*n* all-ones and identity matrices, respectively. Then, by (2), $\bar{A}=J^{\left(n\right)}-I_{n}-A$. Let $j_{n}$ be the all-ones *n*-length column vector, so $j_{n}\;j_{n}^{T}=J^{\left(n\right)}$, and

(126) $$\begin{array}{c} \bar{A}+c_{1}I_{n}+c_{2}J^{\left(n\right)}=\left(c_{1}-1\right)I_{n}+\left(c_{2}+1\right)J^{\left(n\right)}-A, \end{array}$$

for all $c_{1},c_{2}\in R$. Since G is a *d*-regular graph, the largest eigenvalue of A is equal to *d* with $j_{n}$ as an eigenvector. The vector $j_{n}$ is also an eigenvector of $J^{\left(n\right)}$ and $I_{n}$ with eigenvalues *n* and 1, respectively. By (126), and since G is *d*-regular,

(127) $$\begin{array}{c} \left(\bar{A}+c_{1}I_{n}+c_{2}J^{\left(n\right)}\right)\;j_{n}=\left[c_{1}-1+n\left(c_{2}+1\right)-d\right]\;j_{n}, \end{array}$$

which means that $j_{n}$ is an eigenvector of the matrix $\bar{A}+c_{1}I_{n}+c_{2}J^{\left(n\right)}$. The rest n−1 eigenvectors of the symmetric matrix $\bar{A}+c_{1}I_{n}+c_{2}J^{\left(n\right)}$ can be made orthogonal to the eigenvector $j_{n}$ (since eigenvectors of a symmetric matrix, which correspond to distinct eigenvalues, are orthogonal with respect to the standard inner product in $R^{n}$; furthermore, eigenvectors that correspond to the same eigenvalue can be made orthogonal by the Gram-Schmidt procedure). Let v be such an eigenvector, different from $j_{n}$. Then $J^{\left(n\right)}v=j_{n}\left(j_{n}^{T}v\right)=0$, so by (126)

(128) $$\begin{array}{c} \left(\bar{A}+c_{1}I_{n}+c_{2}J^{\left(n\right)}\right)\;v=\left(c_{1}-1\right)v-Av, \end{array}$$

which means that v is also an eigenvector of the adjacency matrix A (since, by assumption, v is an eigenvector of the matrix $\bar{A}+c_{1}I_{n}+c_{2}J^{\left(n\right)}$ in the left-hand side of (128)). The equality Av=λv holds, and $\lambda \in \{\lambda_{2}\left(G\right),\ldots ,\lambda_{n}\left(G\right)\}$ with $\lambda_{2}\left(G\right)$ being the largest among them. This gives that $\lambda \leq \lambda_{2}\left(G\right)$. The symmetric matrix $\bar{A}+c_{1}I_{n}+c_{2}J^{\left(n\right)}$ is positive semi-definite if and only if all its eigenvalues are nonnegative, i.e., (see (127) and (128)),

(129) $$\begin{array}{ccc} & & c_{1}-1+n\left(c_{2}+1\right)-d\geq 0, \end{array}$$

(130) $$\begin{array}{ccc} & & c_{1}-1-\lambda_{2}\left(G\right)\geq 0. \end{array}$$

Setting the two conditions in (129) and (130) to be satisfied with equalities gives

(131) $$\begin{array}{c} c_{1}=1+\lambda_{2}\left(G\right),\;c_{2}=-\frac{n-d+\lambda_{2}\left(G\right)}{n}, \end{array}$$

which implies that (see the left-hand side of (126))

(132) $$\begin{array}{c} \bar{A}+\left(1+\lambda_{2}\left(G\right)\right)\;I_{n}-\left(\frac{n-d+\lambda_{2}\left(G\right)}{n}\right)\;J^{\left(n\right)}⪰0 \end{array}$$

is a positive semi-definite matrix. Clearly, also

(133) $$\begin{array}{c} A-\lambda_{n}\left(G\right)I_{n}⪰0 \end{array}$$

is positive semi-definite. The trace of a product of two *n*-times-*n* positive semi-definite matrices is nonnegative, so it follows from (132) and (133) that

(134) $$\begin{array}{c} \mathrm{tr}\left(\left(\bar{A}+\left(1+\lambda_{2}\left(G\right)\right)\;I_{n}-\left(\frac{n-d+\lambda_{2}\left(G\right)}{n}\right)\;J^{\left(n\right)}\right)\;\left(A-\lambda_{n}\left(G\right)I_{n}\right)\right)\geq 0. \end{array}$$

For an adjacency matrix A of a *d*-regular graph G,

(135) $$\begin{array}{ccc} & & \mathrm{tr}\left(\bar{A}A\right)=\mathrm{tr}\left(A\right)=\mathrm{tr}\left(\bar{A}\right)=0, \end{array}$$

(136) $$\begin{array}{ccc} & & \mathrm{tr}(J^{\left(n\right)}A)=nd, \end{array}$$

and

(137) $$\begin{array}{c} \;\mathrm{tr}\left(I_{n}\right)=\mathrm{tr}\left(J^{\left(n\right)}\right)=n. \end{array}$$

From (134)–(137), expanding the left-hand side in (134) gives the inequality

(138) $$\begin{array}{c} -d\;\left(n-d+\lambda_{2}\left(G\right)\right)-\lambda_{n}\left(G\right)\;\left(d+\left(n-1\right)\lambda_{2}\left(G\right)\right)\geq 0, \end{array}$$

which proves (30) (we have $d+\left(n-1\right)\lambda_{2}\left(G\right)>0$, by the assumption that the *d*-regular graph G is non-complete and non-empty, so $\lambda_{2}\left(G\right)\geq 0$). This alternative proof is next used to find the necessary and sufficient condition for the satisfiability of (30) with equality. An equality in (30) holds if and only if (134) holds with equality (i.e., the trace of the product of the two positive semi-definite matrices in the left-hand sides of (132) and (133) is equal to zero). This holds if and only if the column spaces of the two matrices in the left-hand sides of (132) and (133) are orthogonal. [Clarification: if B,C⪰0 are positive semi-definite matrices, then $B=SS^{T}$ and $C=QQ^{T}$ for some matrices S and Q. Hence, under the assumption that tr(BC)=0,

(139) $$\begin{array}{c} 0=\mathrm{tr}\left(BC\right)=\mathrm{tr}\left(SS^{T}QQ^{T}\right)=\mathrm{tr}\left(S^{T}QQ^{T}S\right)=\mathrm{tr}\left(S^{T}Q\;{\left(S^{T}Q\right)}^{T}\right)=\sum_{i=1}^{n}\sum_{j=1}^{n}{\left(S^{T}Q\right)}_{i,j}^{2} \end{array}$$

implies that $S^{T}Q=0$, so also $BC=S\left(S^{T}Q\right)Q^{T}=0$. Since B and C are symmetric matrices with BC=0, it means that the column spaces of B and C are orthogonal]. These column spaces, however, can be orthogonal only if A has no eigenvalues other than *d*, $\lambda_{2}\left(G\right)$ and $\lambda_{n}\left(G\right)$; otherwise, there is a joint eigenvector of A in the two column spaces of the matrices in the left-hand sides of (132) and (133), so these two column spaces cannot be orthogonal in the latter case. [Clarification: suppose that A has an eigenvector v that corresponds to an eigenvalue $\lambda^{*}\notin \left\{d,\lambda_{2}\left(G\right),\lambda_{n}\left(G\right)\right\}$. Then, by (128) and the value of $c_{1}$ in (131), the right-hand side of (128) is equal to $(\lambda_{2}\left(G\right)-\lambda^{*})v$, and $\left(A-\lambda_{n}\left(G\right)I_{n}\right)v=\left(\lambda^{*}-\lambda_{n}\left(G\right)\right)v$. Both coefficients of v in these two expressions are nonzero, so v belongs to the two column spaces of the two matrices in the left-hand sides of (132) and (133). These column spaces are therefore not orthogonal under the assumption of the existence of a distinct eigenvalue $\lambda^{*}$ of A, as above]. We next distinguish between two cases in regard to the connectivity of the graph G.

(a) If the *d*-regular graph G is connected, then $\lambda_{1}\left(G\right)=d>\lambda_{2}\left(G\right)$. By assumption, G is also non-complete and non-empty graph, so $\lambda_{2}\left(G\right)>\lambda_{n}\left(G\right)$. The connected regular graph G thus has exactly three distinct eigenvalues, so it is strongly regular.
(b) If the *d*-regular graph G is disconnected, then $\lambda_{1}\left(G\right)=d=\lambda_{2}\left(G\right)$. If, by assumption, inequality (30) holds with equality, then $\lambda_{n}\left(G\right)=-1$. This means that G is a disjoint union of equal-sized complete graphs $K_{d+1}$, so it is an imprimitive strongly regular graph (i.e., there are no common neighbors of any pair of non-adjacent vertices in G).

We therefore conclude that, for a non-complete and non-empty *d*-regular graph on *n* vertices, the condition that G is strongly regular is also necessary (and not only sufficient, as it is shown in the first proof) for the inequality in (30) to hold with equality. Equivalently, G being strongly regular is a necessary condition for the inequality in (31) to hold with equality. This completes the proof of the necessity and sufficiency of the condition on the strong regularity of G.

#### 4.2.2. Proof of Corollary 3

Let G be a *d*-regular graph of order *n*, which is non-complete.

*Proof of Item (a)*: By the definition of $g_{2}\left(G\right)$ and $g_{n}\left(G\right)$ in (32), and in light of the equalities $\lambda_{n}\left(\bar{G}\right)=-1-\lambda_{2}\left(G\right)$ and $\lambda_{2}\left(\bar{G}\right)=-1-\lambda_{n}\left(G\right)$, it can be readily verified that the rightmost and leftmost inequalities in (33) are equivalent to (30) and (31), respectively. Furthermore, in light of Corollary 2, each inequality in (33) holds with equality if and only if G is strongly regular.

*Proof of Item (b)*: Let G be a strongly regular graph. We next prove the claim about the dichotomy in the number of distinct values in the sequence ${\left\{g_{\ell }\left(G\right)\right\}}_{\ell =1}^{n}$.

(1) For a *d*-regular graph on *n* vertices, $\lambda_{1}\left(G\right)=d$, and $\lambda_{1}\left(\bar{G}\right)=n-d-1$. Substituting these eigenvalues into (32) gives that $g_{1}\left(G\right)=n-d-2\geq 0$ (G is non-complete, so d≤n−2).
(2) The graph G and its complement $\bar{G}$ are both strongly regular, so each one of them has at most three distinct eigenvalues.
(3) For a strongly regular graph, by Item (a), $g_{2}\left(G\right)=-1=g_{n}\left(G\right)$, and $g_{\ell }\left(G\right)=-1$ if either (i) $\lambda_{\ell }\left(G\right)=\lambda_{2}\left(G\right)$ and $\lambda_{\ell }\left(\bar{G}\right)=\lambda_{2}\left(\bar{G}\right)$, or (ii) $\lambda_{\ell }\left(G\right)=\lambda_{n}\left(G\right)$ and $\lambda_{\ell }\left(\bar{G}\right)=\lambda_{n}\left(\bar{G}\right)$.
(4) By assumption, G is a strongly regular graph, which implies that so is $\bar{G}$. Due to their regularity, $\lambda_{1}\left(G\right)\geq \lambda_{2}\left(G\right)$, and $\lambda_{1}\left(\bar{G}\right)\geq \lambda_{2}\left(\bar{G}\right)$ with equalities, respectively, if and only if G or $\bar{G}$ are disconnected graphs. The sequence ${\left\{g_{\ell }\left(G\right)\right\}}_{\ell =1}^{n}$ gets an additional (third) distinct value if and only if the multiplicities of the smallest and the second-largest eigenvalues of G in the *subsequence* $\left(\lambda_{2}\left(G\right),\ldots ,\lambda_{n}\left(G\right)\right)$ are distinct. Indeed, in the latter case, only one of the following two options is possible: (iii) $\lambda_{\ell }\left(G\right)=\lambda_{2}\left(G\right)$ and $\lambda_{\ell }\left(\bar{G}\right)=\lambda_{n}\left(\bar{G}\right)$, or (iv) $\lambda_{\ell }\left(G\right)=\lambda_{n}\left(G\right)$ and $\lambda_{\ell }\left(\bar{G}\right)=\lambda_{2}\left(\bar{G}\right)$. This holds since, by (4), the multiplicity of the second-largest eigenvalue of G is equal to the multiplicity of the smallest eigenvalue of $\bar{G}$, and similarly, the multiplicity of the smallest eigenvalue of G is equal to the multiplicity of the second-largest eigenvalue of $\bar{G}$. It therefore follows that the third distinct value (as above) is attained by the sequence ${\left\{g_{\ell }\right\}}_{\ell =1}^{n}$ a number of times that is equal to the absolute value of the difference between the multiplicities of the second-largest and the smallest eigenvalues of G in the subsequence $\left(\lambda_{2}\left(G\right),\ldots ,\lambda_{n}\left(G\right)\right)$ (provided that the latter two multiplicities are distinct).

*Proof of Item (c)*: Let G be self-complementary and *d*-regular on *n* vertices. Then,

(140) $$\begin{array}{c} d=\frac{1}{2}\left(n-1\right),\;\lambda_{2}\left(\bar{G}\right)=\lambda_{2}\left(G\right),\;\lambda_{n}\left(\bar{G}\right)=\lambda_{n}\left(G\right). \end{array}$$

Combining the rightmost inequality in (33) and the equalities in (140) readily gives

(141) $$\begin{array}{c} \frac{2\lambda_{2}{\left(G\right)}^{\;2}-\frac{1}{2}\left(n+1\right)}{1+2\lambda_{2}\left(G\right)}\geq -1, \end{array}$$

Since G is non-complete and non-empty, we get $\lambda_{2}\left(G\right)>0$, which then gives from (141) the quadratic inequality

(142) $$\begin{array}{c} 2\lambda_{2}{\left(G\right)}^{\;2}+2\lambda_{2}\left(G\right)-\frac{1}{2}\left(n-1\right)\geq 0. \end{array}$$

Its solution gives (34) (n>1 as otherwise, $G=K_{1}$, but G is by assumption non-complete). Hence, it also follows that

(143) $$\begin{array}{cc} \lambda_{n}\left(G\right) & =\lambda_{n}\left(\bar{G}\right) \end{array}$$

(144) $$\begin{array}{cc} \; & =-1-\lambda_{2}\left(G\right) \end{array}$$

(145) $$\begin{array}{cc} \; & \leq -1-\frac{1}{2}\left(\sqrt{n}-1\right) \end{array}$$

(146) $$\begin{array}{cc} \; & =-\frac{1}{2}\left(\sqrt{n}+1\right), \end{array}$$

where (143) holds since G is (by assumption) self-complementary; (144) holds since G is regular, and (145) holds by (34).

*Proof of Item (d)*: If G is self-complementary and strongly regular, then in light of Items (b) and (c) here, both inequalities in (34) and (35) hold with equality.

#### 4.2.3. Proof of Corollary 4

Let ${\left\{G_{\ell }\right\}}_{\ell \in N}$ be a sequence of regular graphs where $G_{\ell }$ is $d_{\ell }$-regular of order $n_{\ell }$, such that $n_{\ell }\to \infty$ and $\frac{d_{\ell }}{n_{\ell }}\to 0$ as we let *ℓ* tend to infinity. Then,

(147) $$\begin{array}{cc} \underset{\ell \to \infty }{lim sup}\;\omega \left(G_{\ell }\right) & \leq \underset{\ell \to \infty }{lim sup}\;\theta \left({\bar{G}}_{\ell }\right) \end{array}$$

(148) $$\begin{array}{cc} \; & \leq \underset{\ell \to \infty }{lim sup}\;\frac{n_{\ell }\left(1+\lambda_{2}\left(G_{\ell }\right)\right)}{n_{\ell }-d_{\ell }+\lambda_{2}\left(G_{\ell }\right)} \end{array}$$

(149) $$\begin{array}{cc} \; & =1+\underset{\ell \to \infty }{lim sup}\lambda_{2}\left(G_{\ell }\right), \end{array}$$

where (147) holds by the leftmost inequality in (16); (148) holds by the rightmost inequality in (25); (149) holds by the assumption that $n_{\ell }\to \infty$, and since the eigenvalues of $G_{\ell }$ are bounded (in absolute value) by $d_{\ell }$ with $\lim_{\ell \to \infty }\frac{d_{\ell }}{n_{\ell }}=0$. This leads to inequality (40), by a floor operation in the right-hand side of (149), since clique numbers are integers.

We next prove inequality (41). For any graph G with *n* vertices,

(150) $$\begin{array}{c} \alpha (G)\chi (G)\geq n. \end{array}$$

(This well-known inequality holds since the independence number α(G) denotes the size of a largest independent set in G, and in coloring the vertices in G with χ(G) colors, all color classes are independent). Additionally, $\omega \left(G\right)=\alpha \left(\bar{G}\right)$, so

(151) $$\begin{array}{c} \omega \left(G\right)\chi \left(\bar{G}\right)\geq n. \end{array}$$

This gives

(152) $$\begin{array}{cc} \underset{\ell \to \infty }{lim inf}\;\frac{\chi ({\bar{G}}_{\ell })}{n_{\ell }} & \geq \underset{\ell \to \infty }{lim inf}\;\frac{1}{\omega (G_{\ell })} \end{array}$$

(153) $$\begin{array}{cc} \; & =\frac{1}{\underset{\ell \to \infty }{lim sup}\;\omega \left(G_{\ell }\right)} \end{array}$$

(154) $$\begin{array}{cc} \; & \geq \frac{1}{1+\underset{\ell \to \infty }{lim sup}\left\lfloor \lambda_{2}\left(G_{\ell }\right)\right\rfloor }\;, \end{array}$$

where (152) holds by (151); (153) is trivial, and (154) holds by (40).

#### 4.2.4. Proof of Corollary 5

Inequalities (43) and (45) readily follow from Corollary 4 since if ${\left\{G_{\ell }\right\}}_{\ell \in N}$ is a sequence of connected Ramanujan *d*-regular graphs (*d* is a fixed degree of the vertices), then (by definition)

(155) $$\begin{array}{c} \lambda_{2}\left(G_{\ell }\right)\leq 2\sqrt{d-1}, \end{array}$$

for all ℓ∈N. By the assumption that the graph $G_{\ell }$ has order $n_{\ell }$ with $\lim_{\ell \to \infty }n_{\ell }=\infty$, inequalities (43) and (45) are obtained by combining, respectively, (40) and (41) with (155).

Inequality (44) is obtained by combining the leftmost inequality in (24) with (155). Indeed, since $|\lambda_{2}\left(G_{\ell }\right)|\leq d$ (where the degree of the vertices of $G_{\ell }$ is, by assumption, equal to a fixed value *d*), it follows that

(156) $$\begin{array}{c} \theta \left(G_{\ell }\right)\geq \frac{n_{\ell }-2d}{1+\lambda_{2}\left(G_{\ell }\right)}\geq \frac{n_{\ell }-2d}{1+2\sqrt{d-1}}, \end{array}$$

which then yields (44).

#### 4.2.5. Proof of Corollary 6

Let G be a connected Ramanujan *d*-regular graph with *n* vertices. If $G=K_{n}$ is the complete graph, which is a Ramanujan (n−1)-regular graph, then inequality (46) clearly holds (with ω(G)=n). Otherwise, if G is non-complete, then combining the leftmost inequality in (16) and the rightmost inequality in (25) gives that

(157) $$\begin{array}{c} \omega \left(G\right)\leq \left\lfloor \frac{n\left(1+\lambda_{2}\left(G\right)\right)}{n-d+\lambda_{2}\left(G\right)}\right\rfloor , \end{array}$$

where the floor operation in the right-hand side of (157) is enabled because ω(G) is an integer. Since $0\leq \lambda_{2}\left(G\right)\leq 2\sqrt{d-1}$ for a non-complete and *d*-regular connected Ramanujan graph G, and since the function $f_{1}:\left(-1,\infty \right)\to \left(0,\infty \right)$ that is given by

(158) $$\begin{array}{c} f_{1}\left(x\right)≜\frac{n(1+x)}{n-d+x},\;x>-1, \end{array}$$

is monotonically increasing, inequality (46) then follows from (157) and the monotonicity of the function $f_{1}$. Equation (48) follows from (46), (151), and since the chromatic number is an integer.

Inequality (47) holds with equality if $G=K_{n}$ (both sides are equal to 1). Otherwise, inequality (47) holds by the leftmost inequality in (24), since $0\leq \lambda_{2}\left(G\right)\leq 2\sqrt{d-1}$ for a non-complete and *d*-regular Ramanujan graph G, and since the function $f_{2}:\left(-1,\infty \right)\to \left(0,\infty \right)$ that is given by

(159) $$\begin{array}{c} f_{2}\left(x\right)≜\frac{n-d+x}{1+x},\;x>-1, \end{array}$$

is monotonically decreasing.

### 4.3. Proofs for Section 3.3

#### 4.3.1. Proof of Proposition 2

Let $G_{1},\ldots ,G_{k}$ be regular graphs such that, for all ℓ∈[k], the graph $G_{\ell }$ is $d_{\ell }$-regular with $n_{\ell }$ vertices. Let $G=G_{1}⊠\ldots ⊠G_{k}$ be the strong product of these *k* regular graphs. We next prove the two items of Proposition 2.

(a) By the leftmost inequality in (24), unless $G=K_{n}$,
  (160) $$\begin{array}{c} \theta \left(G\right)\geq \frac{n\left(G\right)-d\left(G\right)+\lambda_{2}\left(G\right)}{1+\lambda_{2}\left(G\right)}, \end{array}$$
  where n(G) and d(G) denote, respectively, the order and valency of the strong product, which is a regular graph (since, by assumption, each factor is regular). The following equalities hold as a result of the strong product operation:
  (161) $$\begin{array}{cc} \; & n\left(G\right)=\prod_{\ell =1}^{k}n_{\ell }, \end{array}$$
  (162) $$\begin{array}{cc} \; & d\left(G\right)=\prod_{\ell =1}^{k}\left(1+d_{\ell }\right)-1, \end{array}$$
  (163) $$\begin{array}{cc} \; & \theta \left(G\right)=\prod_{\ell =1}^{k}\theta \left(G_{\ell }\right). \end{array}$$
  Indeed, equality (161) holds since the cardinality of a Cartesian product of finite sets is equal to the product of the cardinalities of each set; equality (162) can be justified by first verifying the special case of a strong product of two regular graphs, and then proceeding by a mathematical induction on *k*. Finally, equality (163) holds by (17) (see Theorem 7 of [6]). Combining the bound in (160) with equalities (161)–(163) gives
  (164) $$\begin{array}{c} \prod_{\ell =1}^{k}\theta \left(G_{\ell }\right)\geq 1+\frac{\prod_{\ell =1}^{k}n_{\ell }-\prod_{\ell =1}^{k}\left(1+d_{\ell }\right)}{1+\lambda_{2}\left(G\right)}. \end{array}$$
  Unless all $G_{\ell }$ (with ℓ∈[k]) are complete graphs, the left-hand side of (164) is strictly larger than 1, and then rearrangement of the terms in (164) gives the lower bound on $\lambda_{2}\left(G\right)$ in (52). Next, the possible loosening of the lower bound in the right-hand side of (52) to the lower bound in the right-hand side of (53) holds by (19) (see Theorem 9 of [6]). Inequality (53) holds with equality if each regular factor $G_{\ell }$ is either edge-transitive (by Theorem 9 of [6]) or strongly regular (by Item (a) of Proposition 1).
(b) Combining (19) with equalities (161)–(163) gives, with n=n(G) and d=d(G),
  (165) $$\begin{array}{cc} \prod_{\ell =1}^{k}\theta \left(G_{\ell }\right) & =\theta (G) \end{array}$$
  (166) $$\begin{array}{cc} \; & \leq -\frac{n\lambda_{n}\left(G\right)}{d-\lambda_{n}\left(G\right)} \end{array}$$
  (167) $$\begin{array}{cc} \; & =-\frac{\prod_{\ell =1}^{k}n_{\ell }\cdot \lambda_{n}\left(G\right)}{\prod_{\ell =1}^{k}\left(1+d_{\ell }\right)-1-\lambda_{n}\left(G\right)}. \end{array}$$
  Unless all $G_{\ell }$ (with ℓ∈[k]) are empty graphs, the denominator in the right-hand side of (167) is strictly positive. This gives (54) after rearrangement of terms. Finally, the transition from (54) to (55) is justified if
  (168) $$\begin{array}{c} \theta \left(G_{\ell }\right)=-\frac{n_{\ell }\lambda_{\min}\left(G_{\ell }\right)}{d_{\ell }-\lambda_{\min}\left(G_{\ell }\right)},\;\forall \;\ell \in \left[k\right]. \end{array}$$
  As above (the end of the proof of Item (a)), the condition in (168) holds if the regular graph $G_{\ell }$ is either edge-transitive or strongly regular.

#### 4.3.2. Proof of Corollary 7

Let G a *d*-regular graph of order *n*. The lower bound on the second-largest eigenvalue $\lambda_{2}\left(G^{⊠\;k}\right)$ in the right-hand side of (59) follows from (52) by setting there $G_{1},\ldots ,G_{k}$ to be all identical to G. The upper bound on the smallest eigenvalue $\lambda_{\min}\left(G^{⊠\;k}\right)$ in the right-hand side of (60) follows in a similar way from (54).

#### 4.3.3. Proof of Proposition 3

Let G be a non-empty and non-complete connected *d*-regular graph on *n* vertices, and let k∈N. Then, $G^{⊠\;k}$ is a connected regular graph, which is non-complete and non-empty (so, its largest eigenvalue is of multiplicity 1). By (59),

(169) $$\begin{array}{c} \lambda_{2}\left(G^{⊠\;k}\right)\geq \frac{n^{k}-{\left(1+d\right)}^{k}}{\theta {\left(G\right)}^{k}-1}-1. \end{array}$$

In order to prove that the *k*-fold strong power $G^{⊠\;k}$ is non-Ramanujan, it is sufficient to show that the lower bound on its second-largest eigenvalue in the right-hand side of (169) is larger than $2\sqrt{d_{k}-1}$; here, $d_{k}={\left(1+d\right)}^{k}-1$ is the valency of the considered strong power (composed of *k* factors, where each factor is the *d*-regular graph G). Since G is *d*-regular and non-complete (i.e., d<n−1 and θ(G)>1), the right-hand side of (169) scales asymptotically like ${\left(\frac{n}{\theta (G)}\right)}^{k}$ (for a sufficiently large *k*), whereas the expression $2\sqrt{d_{k}-1}$ scales asymptotically like $2{\left(1+d\right)}^{\frac{k}{2}}$. Comparing these two exponents gives that if

(170) $$\begin{array}{c} \theta \left(G\right)<\frac{n}{\sqrt{1+d}}, \end{array}$$

then the exponential growth rate of the right-hand side of (169) is larger than that one of $2\sqrt{d_{k}-1}$. Hence, for sufficiently large *k*, the strong power $G^{⊠\;k}$ is a (highly) non-Ramanujan graph under the condition in (170). This means that there exists $k_{0}\in N$ such that the strong power $G^{⊠\;k}$ is non-Ramanujan for all $k\geq k_{0}$. We next obtain an explicit value of such $k_{0}$, which is not necessarily the smallest one, proving that such a valid value for $k_{0}$ is given by (63). To that end, based on the above explanation, one needs to deal with the inequality

(171) $$\begin{array}{c} \frac{n^{k}-{\left(1+d\right)}^{k}}{\theta {\left(G\right)}^{k}-1}-1>2\sqrt{{\left(1+d\right)}^{k}-2}. \end{array}$$

In order to obtain a closed-form solution, we strengthen the condition in (171) to

(172) $$\begin{array}{c} \frac{n^{k}-{\left(1+d\right)}^{k}}{\theta {\left(G\right)}^{k}}-1\geq 2{\left(1+d\right)}^{\frac{k}{2}}. \end{array}$$

Dividing both sides of (172) by ${\left(1+d\right)}^{\frac{k}{2}}$ gives

(173) $$\begin{array}{c} {\left(\frac{n}{\sqrt{1+d}\;\theta \left(G\right)}\right)}^{k}-{\left(\frac{\sqrt{1+d}}{\theta (G)}\right)}^{k}-{\left(1+d\right)}^{-\frac{k}{2}}\geq 2. \end{array}$$

Let k≥3. The condition imposed in (173) can be further strengthened to

(174) $$\begin{array}{c} {\left(\frac{n}{\sqrt{1+d}\;\theta \left(G\right)}\right)}^{k}-{\left(\frac{\sqrt{1+d}}{\theta (G)}\right)}^{k}\geq 2+{\left(1+d\right)}^{-\frac{3}{2}}. \end{array}$$

Since d<n−1 for a non-complete *d*-regular graph of order *n*, for all k≥3,

(175) $$\begin{array}{cc} {\left(\frac{n}{\sqrt{1+d}\;\theta \left(G\right)}\right)}^{k}-{\left(\frac{\sqrt{1+d}}{\theta (G)}\right)}^{k}\; & ={\left(\frac{n}{\sqrt{1+d}\;\theta \left(G\right)}\right)}^{k}\;\left[1-{\left(\frac{1+d}{n}\right)}^{k}\right]\\ \; & \geq {\left(\frac{n}{\sqrt{1+d}\;\theta \left(G\right)}\right)}^{k}\;\left[1-{\left(\frac{1+d}{n}\right)}^{3}\right]>0, \end{array}$$

which, by combining (174) and (175), gives the stronger condition

(176) $$\begin{array}{c} \frac{n^{3}-{\left(1+d\right)}^{3}}{n^{3}}\;{\left(\frac{n}{\sqrt{1+d}\;\theta \left(G\right)}\right)}^{k}\geq 2+{\left(1+d\right)}^{-\frac{3}{2}}, \end{array}$$

with k≥3. Solving inequality (176) implies that inequality (171) is satisfied for all $k\geq k_{0}$, with the closed-form expression of $k_{0}$ in (63). It therefore gives that if G is a *d*-regular graph on *n* vertices, which satisfies the condition in (170), then $G^{⊠\;k}$ is non-Ramanujan for all $k\geq k_{0}$ (it becomes, in fact, a highly non-Ramanujan graph since both sides of inequality (171) have different exponential growth rates, so the condition for a Ramanujan graph is strongly violated for the strong power $G^{⊠\;k}$ when the value of *k* is increased).

We next specialize this result for graphs that are self-complementary and vertex-transitive. For n=1, the complete graph $G=K_{1}$ is a self-complementary and vertex-transitive graph, whose all strong powers are also isomorphic to $K_{1}$, so they are therefore non-Ramanujan graphs.

Let G be a graph of order n>1 that is self-complementary and vertex-transitive, so it is *d*-regular with $d=\frac{1}{2}\left(n-1\right)$. Additionally, for such a graph G, the Lovász θ-function is equal to $\theta \left(G\right)=\sqrt{n}$, and it coincides with the Shannon capacity of G (see Theorems 8 and 12 of [6]). Then,

(177) $$\begin{array}{c} \frac{n}{\sqrt{d+1}}=\sqrt{\frac{2n^{2}}{n+1}}>\sqrt{n}=\theta \left(G\right), \end{array}$$

so the required condition in Proposition 3 is fulfilled by graphs of order *n* that are self-complementary and vertex-transitive. The value of $k_{0}$ in (63) is specialized for such graphs to

(178) $$\begin{array}{cc} k_{0} & =\max\left\{3,\left\lceil \frac{2\log\left(\frac{8n^{3}}{8n^{3}-{\left(n+1\right)}^{3}}\right)+2\log\left(2+\sqrt{\frac{8}{{\left(n+1\right)}^{3}}}\;\right)}{\log\left(\frac{2n}{n+1}\right)}\right\rceil \right\} \end{array}$$

(179) $$=\{\begin{array}{cc} 5 & \mathrm{if}\;n=5,\\ 4 & \mathrm{if}\;n=9,\\ 3 & \mathrm{if}\;n\geq 13\;\mathrm{with}\;n\equiv 1\;(\mathrm{mod}\;4)., \end{array}$$

The constraint on *n* in (179) is the necessary condition on *n* in Remark 10.

### 4.4. Proofs for Section 3.4

#### 4.4.1. Proof of Proposition 4

(a) Let $G_{1},\ldots ,G_{k}$ be *k* simple, finite and undirected graphs, $\left|V(G_{\ell })\right|=n_{\ell }$ for ℓ∈[k], and let $G=G_{1}⊠\ldots ⊠G_{k}$. We provide two alternative simple proofs of (65).
  *First proof:*
  (180) $$\begin{array}{cc} \chi (G) & \geq \theta (\bar{G}) \end{array}$$
  (181) $$\begin{array}{cc} \; & \geq \frac{\left|V(G)\right|}{\theta (G)} \end{array}$$
  (182) $$\begin{array}{cc} \; & =\prod_{\ell =1}^{k}\frac{\left|V(G_{\ell })\right|}{\theta (G_{\ell })}, \end{array}$$
  where (180) holds by (16), (181) holds by (18), and equality (182) holds by (17) and since $V\left(G\right)=V\left(G_{1}\right)\times \ldots \times V\left(G_{k}\right)$. The ceiling operation can be add to the right-hand side of (182) since a chromatic number is an integer.
  *Second proof:*
  (183) $$\begin{array}{cc} \chi (G) & \geq \frac{\left|V(G)\right|}{\alpha (G)} \end{array}$$
  (184) $$\begin{array}{cc} \; & \geq \frac{\left|V(G)\right|}{\theta (G)} \end{array}$$
  (185) $$\begin{array}{cc} \; & =\prod_{\ell =1}^{k}\frac{\left|V(G_{\ell })\right|}{\theta (G_{\ell })}, \end{array}$$
  where (183) holds by (150), (184) holds by (23), and (185) is (182).
  We next prove (66).
  (186) $$\begin{array}{cc} \chi (\bar{G}) & \geq \theta (G) \end{array}$$
  (187) $$\begin{array}{cc} \; & =\prod_{\ell =1}^{k}\theta \left(G_{\ell }\right), \end{array}$$
  where (186) holds by (15), and (187) holds by (17).
(b) Let $G_{1},\ldots ,G_{k}$ be regular graphs, where $G_{\ell }$ is $d_{\ell }$-regular of order $n_{\ell }$ for all ℓ∈[k]. Inequality (67) is (65). Inequality (68) follows from (19) and (67). Furthermore, by Item (a) in Proposition 1, inequality (68) holds with equality if each regular graph $G_{\ell }$, for ℓ∈[k], is either edge-transitive or strongly regular.
(c) By (182), with $|V\left(G_{\ell }\right)|=n_{\ell }$,
  (188) $$\begin{array}{c} \frac{\left|V(G)\right|}{\theta (G)}=\prod_{\ell =1}^{k}\frac{n_{\ell }}{\theta (G_{\ell })}. \end{array}$$
  Suppose that, for all ℓ∈[k], $G_{\ell }$ is $d_{\ell }$-regular, and it is also either edge-transitive or strongly regular. By Item (a) in Proposition 1, for all ℓ∈[k],
  (189) $$\begin{array}{c} \theta \left(G_{\ell }\right)=-\frac{n_{\ell }\;\lambda_{\min}\left(G_{\ell }\right)}{d_{\ell }-\lambda_{\min}\left(G_{\ell }\right)}. \end{array}$$
  Combining (188) and (189) gives
  (190) $$\begin{array}{c} \frac{\left|V(G)\right|}{\theta (G)}=\prod_{\ell =1}^{k}\left(1-\frac{d_{\ell }}{\lambda_{\min}\left(G_{\ell }\right)}\right). \end{array}$$
  On the other hand, since $G=G_{1}⊠\ldots ⊠G_{\ell }$ is *d*-regular, with d≜d(G) as given in (70), it follows from (19) that
  (191) $$\begin{array}{c} \theta \left(G\right)\leq -\frac{|V\left(G\right)|\;\lambda_{\min}\left(G\right)}{d\left(G\right)-\lambda_{\min}\left(G\right)}. \end{array}$$
  It should be noted, in regard to (191), that even if all $G_{\ell }$’s are regular and edge-transitive graphs, their strong product G is not necessarily edge-transitive. In fact, G is not edge-transitive, unless all the *k* factors ${\left\{G_{\ell }\right\}}_{\ell =1}^{k}$ are complete graphs (see Theorem 3.1 of [28]). For this reason, (191) does not hold in general with equality (see Theorem 9 of [6]). Finally, combing (190) and (191) gives inequality (69).
(d) Let $G_{1},\ldots ,G_{k}$ be regular graphs, where $G_{\ell }$ is $d_{\ell }$-regular on $n_{\ell }$ vertices for all ℓ∈[k]. Then, under the assumptions of Item (d),
  (1) (192) $$\prod_{\ell =1}^{k}\frac{\left|V(G_{\ell })\right|}{\theta (G_{\ell })}=\prod_{\ell =1}^{k}\theta \left(\bar{G_{\ell }}\right)$$
    (193) $$\begin{array}{cc} \; & \geq \prod_{\ell =1}^{k}\omega \left(G_{\ell }\right) \end{array}$$
    where (192) holds since, by assumption, each of the graphs $G_{1},\ldots ,G_{k}$ is vertex-transitive or a strongly regular graph (this is because Theorem 8 of [6] and equality (27) provide different sufficient conditions for inequality (18) to hold with equality). Inequality (193) holds by the leftmost inequality in (16).
  (2) (194) $$\prod_{\ell =1}^{k}\left(1-\frac{d_{\ell }}{\lambda_{\min}\left(G_{\ell }\right)}\right)=\prod_{\ell =1}^{k}\frac{\left|V(G_{\ell })\right|}{\theta (G_{\ell })}$$
    (195) $$\begin{array}{cc} \; & \geq \prod_{\ell =1}^{k}\omega \left(G_{\ell }\right) \end{array}$$
    where, by Item (a) of Proposition 1, equality (194) holds since (by assumption), for all ℓ∈[k], the graph $G_{\ell }$ is either regular and edge-transitive, or a strongly regular graph. Inequality (195) holds under the same reasoning as of (192) and (193).
  To summarize, it shows that under proper assumptions, the lower bound on the chromatic number of G in the right-hand side of (65), or even its loosened bound in the right-hand side of (68), are larger than or equal to the lower bound $\prod_{\ell =1}^{k}\omega \left(G_{\ell }\right)$.
(e) By (66),
  (196) $$\begin{array}{c} \chi \left(\bar{G}\right)\geq \prod_{\ell =1}^{k}\theta \left(G_{\ell }\right). \end{array}$$
  Let, for all ℓ∈[k], the graph $G_{\ell }$ be $d_{\ell }$-regular on $n_{\ell }$ vertices, and suppose that it is either edge-transitive or strongly regular. Then, by Item (a) of Proposition 1,
  (197) $$\begin{array}{c} \theta \left(G_{\ell }\right)=-\frac{n_{\ell }\;\lambda_{\min}\left(G_{\ell }\right)}{d_{\ell }-\lambda_{\min}\left(G_{\ell }\right)},\;\forall \;\ell \in \left[k\right]. \end{array}$$
  Combining (196) and (197), followed by taking a ceiling operation on the lower bound on the chromatic number $\chi (\bar{G})$, gives (71).
(f) By the assumption that $G_{1},\ldots ,G_{k}$ are self-complementary,
  (198) $$\begin{array}{c} \theta \left(G_{\ell }\right)=\theta \left({\bar{G}}_{\ell }\right),\;\forall \;\ell \in \left[k\right]. \end{array}$$
  Furthermore, by the assumption that for all ℓ∈[k], $G_{\ell }$ is a graph on $n_{\ell }$ vertices that is either vertex-transitive or strongly regular,
  (199) $$\begin{array}{c} \theta \left(G_{\ell }\right)\;\theta \left({\bar{G}}_{\ell }\right)=n_{\ell },\;\forall \;\ell \in \left[k\right]. \end{array}$$
  Combining (198) and (199) gives
  (200) $$\begin{array}{c} \theta \left(G_{\ell }\right)=\sqrt{n_{\ell }},\;\forall \;\ell \in \left[k\right]. \end{array}$$
  Consequently, by (65) and (200),
  (201) $$\begin{array}{cc} \chi (G) & \geq \left\lceil \prod_{\ell =1}^{k}\frac{n_{\ell }}{\theta (G_{\ell })}\right\rceil \end{array}$$
  (202) $$\begin{array}{cc} \; & =\left\lceil \prod_{\ell =1}^{k}\sqrt{n_{\ell }}\;\right\rceil \end{array}$$
  (203) $$\begin{array}{cc} \; & =\left\lceil \sqrt{n}\;\right\rceil , \end{array}$$
  and, from (66) and (200),
  (204) $$\begin{array}{cc} \chi (\bar{G}) & \geq \left\lceil \prod_{\ell =1}^{k}\theta \left(G_{\ell }\right)\right\rceil \end{array}$$
  (205) $$\begin{array}{cc} \; & =\left\lceil \prod_{\ell =1}^{k}\sqrt{n_{\ell }}\;\right\rceil \end{array}$$
  (206) $$\begin{array}{cc} \; & =\left\lceil \sqrt{n}\;\right\rceil . \end{array}$$
  This proves (72) and (73), and it completes the proof of Proposition 4.

#### 4.4.2. Proof of Corollary 8

The rightmost inequality in (78) is a well-known upper bound on the chromatic number of strong products (see [2] or Theorem 3 of [19]). The leftmost inequality in (78) gives a lower bound on the chromatic number of a strong product of (not necessarily distinct) non-complete, and strongly regular graphs. It readily follows by combining equality (26) in Corollary 1, together with inequality (65) in Proposition 4. Finally, by Part 1 of Item (d) in Proposition 4, the leftmost term in (78) is larger than or equal to the product of the clique numbers of ${\left\{G_{\ell }\right\}}_{\ell =1}^{k}$.

## Data Availability

Not applicable.
